# Targeting uPAR with an antibody-drug conjugate suppresses tumor growth and reshapes the immune landscape in pancreatic cancer models

**DOI:** 10.1126/sciadv.adq0513

**Published:** 2025-01-17

**Authors:** Virginia Metrangolo, Michaela Hansen Blomquist, Ananya Dutta, Henrik Gårdsvoll, Oliver Krigslund, Kirstine Sandal Nørregaard, Henrik Jessen Jürgensen, Michael Ploug, Matthew J. Flick, Niels Behrendt, Lars H. Engelholm

**Affiliations:** ^1^ The Finsen Laboratory, Rigshospitalet, DK-2200 Copenhagen, Denmark.; ^2^ Biotech Research & Innovation Centre (BRIC), Faculty of Health and Medical Sciences, University of Copenhagen, DK-2200 Copenhagen, Denmark.; ^3^ Department of Medicine, Duke University, Durham, NC 27710, USA.; ^4^ Department of Medicine and the UNC Blood Research Center, University of North Carolina, Chapel Hill, NC 27599, USA.; ^5^ Department of Clinical Medicine, Faculty of Health and Medical Sciences, University of Copenhagen, GK-2200 Copenhagen, Denmark.

## Abstract

Antibody-drug conjugates (ADCs) hold promise to advance targeted therapy of pancreatic ductal adenocarcinoma (PDAC), where the desmoplastic tumor stroma challenges effective treatment. Here, we explored the urokinase plasminogen activator receptor (uPAR) as a candidate ADC target in PDAC, harnessing its massive tumoral and stromal expression in this stroma-dense tumor. We generated a site-specific ADC offering high-affinity, cross-species reactivity, and efficient internalization of the anti-uPAR monoclonal antibody, FL1, carrying a potent anthracycline derivative (PNU-158692). In vitro, FL1-PNU exhibited potent and specific cytotoxicity against uPAR-expressing PDAC cell lines, stromal and immune cells, and bystander killing of uPAR-negative cells. In vivo, the ADC induced remission or sustained tumor regression and extended survival in xenograft models. In syngeneic orthotopic models, the antitumor effect promoted immunomodulation by enhancing infiltrating immune effectors and decreasing immunosuppressive cells. This study lays grounds for further exploring FL1-PNU as a putative clinical ADC candidate, potentially providing a promising therapeutic avenue for PDAC as a monotherapy or in combinatorial regimens.

## Teaser

Targeting uPAR with an ADC suppresses tumor growth and reshapes the immune landscape in pancreatic cancer models.

## INTRODUCTION

Pancreatic ductal adenocarcinoma, PDAC, is one of the deadliest forms of cancer, with a steadily increasing incidence and the poorest 5-year survival rate in over 40 years (<10%) (*1*, *2*). This dismal scenario stems from a combination of diagnostic and therapeutic challenges. At the time of diagnosis, most patients with PDAC (~80%) present with advanced or metastatic tumors and are not eligible for surgical resection, currently the only potentially curative approach. Thus, these patients receive palliative chemotherapy with FOLFIRINOX or gemcitabine plus nab-paclitaxel as first-line therapy, which usually shows low response rates and leads to marginal survival improvements (*1*, *2*). The remaining 20% of patients who undergo tumor resection and adjuvant chemotherapy may eventually relapse likely because of micrometastases present at the time of surgery (*3*). One decisive roadblock to effective PDAC therapy is the desmoplastic and immunosuppressive tumor stroma (*4*). This unique architecture impedes intratumoral drug delivery and infiltration of anticancer immune cells, with consequent drug and immune resistance (*4*). Accordingly, the use of immunotherapies (ITs), like monoclonal antibodies (mAbs) and derivatives (e.g., immune checkpoint inhibitors (ICIs), CAR-T cells, or bispecific mAbs), which have proven success in some cancer types, have been somewhat discouraging in patients with PDAC, underscoring the urgent need for more effective treatment modalities (*4*–*6*).

Antibody-drug conjugates (ADCs) that deliver a targeted cytotoxic insult via drug-loaded tumor-homing mAbs have shown robust proof of concept in various solid tumors, as evidenced by the recent approvals of six new agents in only 4 years—reaching a total of 13 Food and Drug Administration–approved ADC products in 2023 (*6*, *7*). The remarkable clinical success of ADCs in these cancers holds promise for tackling the challenging PDAC biology (*1*, *8*). Compared to functional antagonist approaches, ADCs can elicit orthogonal killing mechanisms, like bystander cell cytotoxicity (*6*, *7*, *9*) or immunogenic cell death (*6*, *7*, *10*) with the potential to amplify the overall antitumor response by alleviating constraints of target heterogeneity and immunosuppression. Despite intensive research, only a few tumor-associated surface antigens (Ags) have been investigated as ADC targets in PDAC, but no approved agents currently exist for these patients (*1*, *8*).

In this work, we explored the urokinase plasminogen activator receptor, uPAR, as a potential ADC target for PDAC therapy. Owing to its aberrant overexpression and recognized role in the progression of most aggressive cancers, PDAC included, uPAR has been pursued as a therapeutic target for almost four decades (*11*, *12*). Although most attempts with traditional inhibitory strategies have been ineffective, the therapeutic landscape targeting this receptor has resurged with the emergence of various targeted cytotoxic interventions harnessing its pronounced tumor-selective expression (*11*).

Notably, PDAC exhibits the highest uPAR mRNA levels over all other tumor types and a strongly differential expression from normal pancreas and chronic pancreatitis (*11*–*13*). Yet, in contrast to clinically relevant targets like HER2 or MUC-1, uPAR is also abundant in various noncancerous cells present in the activated tumor-supporting stroma, including cancer-associated fibroblasts (CAFs), macrophages, neutrophils, and endothelial cells (*11*). This simultaneous cancer and stromal expression might provide a superior therapeutic gain in PDAC and likewise stroma-rich tumors by facilitating targeted and orthogonal antitumor activities. In addition, the genetic stability of the stroma may also limit the development of drug resistance (*4*). The rationale for uPAR-mediated stromal targeting is supported by preclinical examples of nanoparticles (NPs) and oncolytic viruses (OVs) (*11*, *14*). However, these approaches are still far from achieving clinical impact in PDAC and have inherent limitations compared to ADCs. Examples of these include the larger size and inability to mediate effector mechanisms for NPs (*15*) and complex administration routes and immunogenicity in the case of OVs (*16*).

Here, we describe the generation and preclinical validation of a novel uPAR-specific ADC, FL1-PNU, as a potential therapeutic candidate for PDAC. We designed our ADC to include (i) a high-affinity, internalizing, and species cross-reactive anti-uPAR mAb, FL1, and (ii) a potent anthracycline payload (PNU-159682) site-specifically linked to FL1 by a lysosomally cleavable linker. This ADC demonstrated potent and specific antitumor activity in various in vitro and in vivo models, including xenograft and syngeneic allograft PDAC models. In the latter, the antitumor effect was shown to reshape the suppressive tumor microenvironment (TME) by promoting local immunomodulation. Besides validating uPAR as a promising ADC target in PDAC, these studies provide proof of efficacy of this ADC strategy and rationale for advancing its investigation either as monotherapy or in combination with standard of care or other ITs.

## RESULTS

### Selecting efficiently internalizing anti-uPAR mAb candidates for ADC development

Efficient antibody uptake is a prerequisite for targeted delivery of cytotoxic drugs to receptor-positive cancer cells via internalizing ADCs. Therefore, to identify optimal candidate(s) for our uPAR-targeting ADC, we focused on evaluating the internalization performance of a panel of murine anti-uPAR immunoglobulin G1 (IgG1) mAbs. Their binding properties, including epitope and species specificity, and their ability to inhibit uPA binding, are detailed in the Supplementary Materials, table S1 and fig. S1, respectively. All mAbs display high affinity toward human uPAR (huPAR), with dissociation constant values ranging between 20 pM and 0.7 nM, qualifying them for the screening process.

**Fig. 1.**
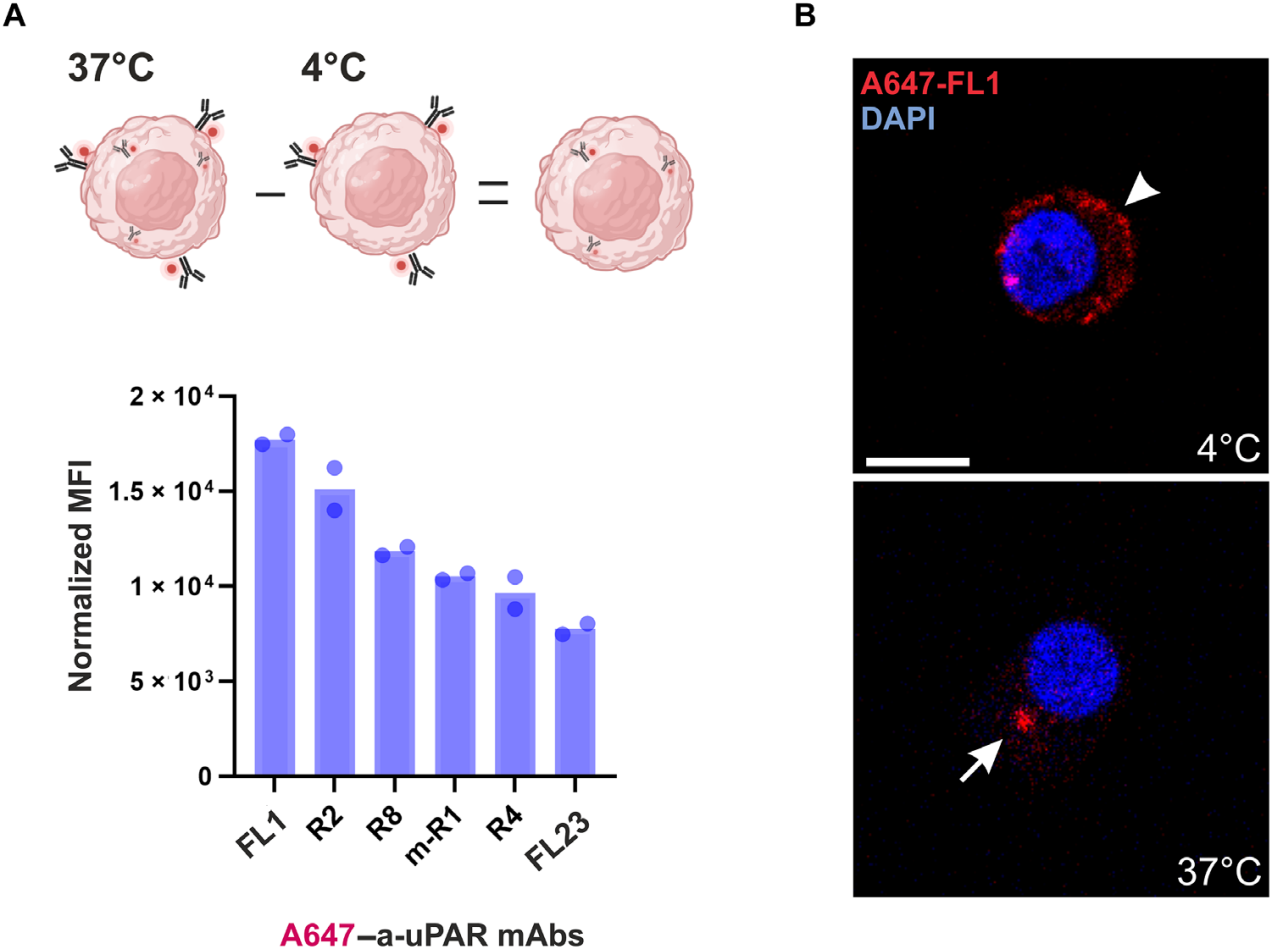
Cellular uptake of anti-uPAR mAbs in uPAR-positive U937 cells. (**A**) Top, experimental setup for calculating internalization efficiency. Bottom, histograms representing internalized A647–a-uPAR mAbs by U937 cells, as quantified by flow cytometry, after 4 hours of incubation at 37°C with 5 μg/ml of each conjugate. Internalized fluorescence is expressed as mean fluorescence intensity (MFI) (total fluorescence at 37°C–surface-bound signal at 4°C). This value was corrected for small variations in the degree of labeling (DOL) of the various mAbs. Shown are the data for two independent experiments. (**B**) Representative confocal microscopy images highlighting (i) the surface localization of Alexa 647–FL1 in U937 cells (in red) after incubation at 4°C and (ii) the internalized fraction after incubation for 4 hours at 37°C (punctuated intracellular structures). Cell nuclei were counterstained with 4′,6-diamidino-2-phenylindole (DAPI, blue). Scale bar, 20 μm.

We evaluated the uptake efficiency of the panel by flow cytometry using a reference uPAR-positive leukemic cell line, U937, and Alexa 647 conjugates of the mAbs (referred to as A647-mAbs). Cells were incubated with the A647-a-uPAR mAbs for 4 hours at 37°C, while a parallel sample was maintained at 4°C, serving as a “no internalization” control (Fig. 1A, top). After incubation, intracellular fractions were calculated as the difference between the total fluorescence detected at 37°C (surface + internalized) and the membrane-bound fraction at 4°C. Most antibodies displayed appreciable intracellular uptake, with FL1 exhibiting the highest internalization capacity (Fig. 1A, bottom). FL1 was raised by immunizing uPAR-deficient mice with recombinant soluble human uPAR (huPAR) and was found to bind both the human and murine uPAR (muPAR) with high affinity (fig. S1 and table S1). Cellular uptake of FL1 was also confirmed by confocal immunofluorescence microscopy (Fig. 1B). On the basis of its internalization properties and cross-species reactivity, we selected FL1 as the lead candidate for further development as an ADC delivery vehicle.

### FL1 internalizes and traffics to the lysosome after binding uPAR on PDAC cells

**Fig. 2.**
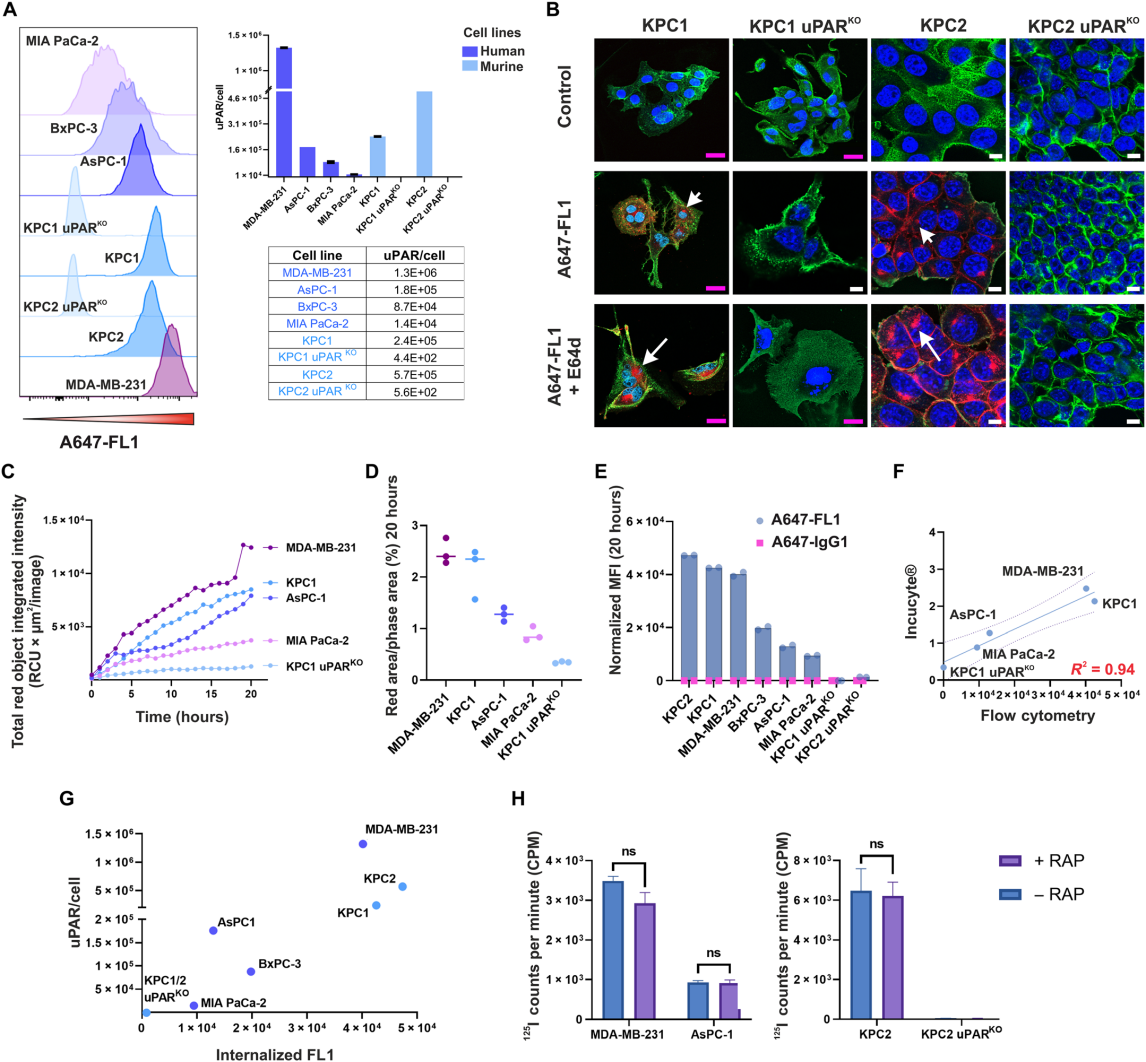
Binding of FL1 to uPAR initiates internalization and lysosomal routing in PDAC cells through an LRP1-independent mechanism. (**A**) Left, representative flow cytometry histograms showing specific binding of A647-coupled FL1 to uPAR-expressing cells. Right, number of receptor binding sites per cell, estimated using QSC beads (*n* = 3). Numbers below 1 × 10^3^ reflect a lack of uPAR expression. (**B**) Confocal microscopy analysis showing uPAR-dependent FL1 uptake and lysosomal trafficking in murine receptor–positive KPC cells. Cells were incubated for 4 hours at 37°C with A647-labeled mAb in the absence or presence of E64d. No uptake is observed in uPAR^KO^ cells. Cell membranes, in green, and nuclei, in blue, were stained with Alexa 488-labeled wheat germ agglutinin and DAPI, respectively. Scale bar, (pink) 20 μm and (white) 10 μm. (**C**) Real-time monitoring of FL1 uptake using the Incucyte S3 live-cell analysis system. The red fluorescent intensity shows a clear time-dependent increase in the uPAR-positive cells (*n* = 3). (**D**) Scatter plot of total uptake at 20 hours normalized to the phase area (cell confluence) (*n* = 3). (**E**) Flow cytometry analysis of A647-labeled FL1 internalization in the entire PDAC cell panel after 20 hours of incubation at 37°C with A647-FL1 and isotype control mAb, A647-IgG1. After incubation, surface-bound mAbs were removed by proteolytic treatment with a trypsin-EDTA, proteinase K solution (*n* = 2). (**F**) Correlation between flow cytometry and Incucyte live-imaging data. The regression line (blue) and the R-squared value (red) are displayed. (**G**) Scatterplot illustrating the relationship between detected cellular uPAR receptor number (*y* axis) and corresponding FL1 internalization (*x* axis). (**H**) Histograms showing internalized ^125^I labeled FL1 in selected human (left) and murine (right) uPAR-expressing cells in the presence and absence of 500 nM RAP (*n* = 3). All data are representative of at least two independent experiments.

We next examined FL1 binding to uPAR and subsequent uptake in a panel of established PDAC cell lines, respectively, human AsPC-1, BxPC-2, and MIA PaCa-2 cells, and two murine KPC (K-ras^LSL.G12D/+^; p53^LSL.R172H/+^; Elas-CreER)–derived cell lines, KPC1 and KPC2 cells. As positive and negative controls, we included the high-uPAR expressing triple-negative breast cancer MDA-MB-231 cells and two uPAR-knockout (KO) KPC cell lines derived from the parental KPC cells through CRISPR RNA–guided Fokl nuclease–mediated gene editing (*17*). To determine the number of surface-exposed uPAR molecules that were detectable by FL1, we performed quantitative flow cytometry using the A647-labeled mAb and anti-mouse IgG Quantum Simply Cellular (QSC) calibration beads (*18*, *19*), as outlined in Material and Methods. We found a variable number of uPAR molecules across our PDAC models (Fig. 2A). As expected, no measurable levels were detected in the two uPAR-deficient KPC cell lines (KPC1^KO^ and KPC2^KO^), while 1.3 × 10^6^ receptors per cell were determined on MDA-MB-231 cells, consistent with other reports (*20*). These data confirmed that the FL1 binding epitope is available for targeting on both huPAR- and muPAR-expressing cells and validated FL1 binding specificity. Orthogonal mRNA and protein analyses yielded a comparable ranking of receptor expression, further corroborating these results (fig. S2).

To investigate the internalization properties of FL1 in these cells, we used various approaches. A qualitative immunofluorescence staining was conducted in the two matching pairs of KPC and KPC uPAR^KO^ cell lines, followed by confocal microscopy (Fig. 2B). After 4 hours of incubation at 37°C with A647-labeled FL1, distinct red intracellular puncta appeared throughout the cytosol of both parental KPC cell lines, indicating antibody uptake and suggesting routing to the lysosomes, consistent with the findings in U937 cells. Membrane-bound fluorescence revealed a residual amount of noninternalized FL1 on the surface of KPC2 cells. Lysosomal trafficking of FL1 was confirmed by preincubating the cells with the lysosomal protease inhibitor E64d before adding FL1. E64d impairs antibody clearance by lysosomal sulfhydryl proteases, leading to accumulation (*21*). Accordingly, we observed a more pronounced punctate signal in both KPC cell lines. No fluorescence was detected in the corresponding uPAR^KO^ cells under any of the conditions tested.

To quantify the time course of FL1 uptake, we used live-cell imaging. This study included five cell lines that were classified as high (MDA-MB-231, KPC1, and AsPC-1), low (MIA PaCa-2), or nonexpressing (KPC1 uPAR^KO^) based on their uPAR expression levels. Internalization was monitored in real time using the Incucyte Live-Cell Analysis system with FL1 coupled to a pH-sensitive red label (Incucyte Red Fabfluor-pH dye). This allowed us to visualize and measure the mAb trafficking into the acidic endosomes and lysosomes. The time course analysis of the detected red fluorescence intensity over 20 hours of incubation is shown in Fig. 2C, while fig. S3 displays a complete inventory of end point analyses for all cell lines with images acquired at 20 hours.

An accumulation of red fluorescence was recorded over time in all uPAR-positive cells, while no uptake was observed in the uPAR^KO^ cells (Fig. 2C). This observation corroborated that FL1 effectively internalizes upon uPAR binding, subsequently routing to the lysosomal compartment. Total uptake at 20 hours of incubation was normalized to the relative cell confluence (phase area) to correct for cell growth differences (Fig. 2D). To use an orthogonal method and broaden the analysis to the entire cell panel, we used flow cytometry and measured the total mAb uptake after 20 hours of incubation with A647-labeled FL1 and a nonbinding isotype control, A647-labeled IgG1 (Fig. 2E). These results correlated strongly with the imaging data, thus providing independent validation of the specific internalization of FL1 by uPAR (Fig. 2F).

Furthermore, these studies also allowed us to evaluate FL1 uptake relative to the receptor surface density in the investigated cells. Although some degree of correlation was indeed found between these parameters (Fig. 2G), it was clear that even cell lines with low/moderate expression levels of uPAR, like MIA PaCa-2 or BxPC-3, showed a substantial internalization of FL1 (Fig. 2G).

We next explored the mechanism underlying the uPAR-mediated internalization of FL1, as this is an essential step in the ADC mechanism of action (MOA). Two main processes are known to regulate intracellular trafficking of uPAR. The first involves a clathrin-dependent endocytic route that mediates the clearance of the uPAR•uPA•PAI-1 complexes following association with the LRP1 (low-density lipoprotein receptor–related protein 1) (*22*). The second mechanism consists of a ligand- and LRP1-independent macropynocytic-like process, which regulates constitutive endocytosis and recycling of uPAR (*23*). As shown in Fig. 2H, in the presence of the specific LRP1 antagonist RAP (receptor-associated protein), the uptake levels of FL1 were not significantly perturbed in any of the analyzed human and murine cell lines. These data suggest that an LRP1-independent mechanism mediates the uptake of FL1-uPAR complexes in these cells. Together, these studies provided robust evidence for an FL1-induced internalization of uPAR, supposedly via receptor clustering, and subsequent lysosomal routing in PDAC cells.

### FL1- PNU exerts potent and uPAR-dependent cytotoxicity in PDAC cell lines

To successfully target therapy-resistant malignancies like PDAC, a strong cytotoxic insult is required. Therefore, we selected PNU-159682, a highly potent anthracycline derivative (hereafter referred to as PNU), which recently emerged in the ADC landscape (*24*), as the cytotoxic payload of our a-uPAR ADC.

To assess the cytotoxicity of PNU on our PDAC cell panel, we conducted a 72-hour treatment with free toxin in the concentration range of 5 pM to 70 nM and then measured cell viability via the 3-(4,5-dimethylthiazol-2-yl)-5-(3-carboxymethoxyphenyl)-2-(4-sulfo phenyl)-2H-tetrazolium (MTS) assay. Confirming the strong cytotoxic potency of this payload, we observed that a concentration of PNU as low as 5 pM eradicated all cells in all examined cell lines (fig. S4). In contrast, unmodified FL1 had no antiproliferative effect per se.

**Fig. 3.**
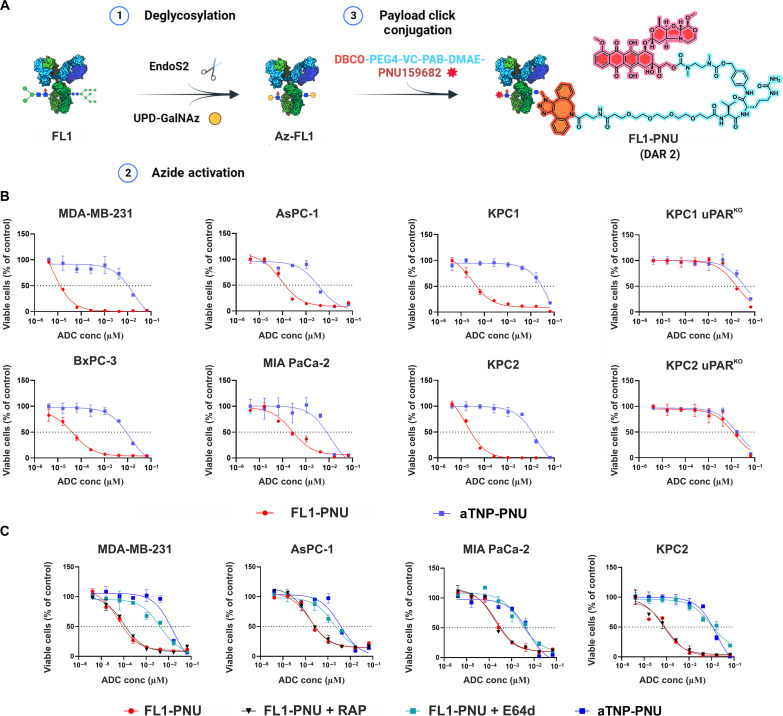
In vitro cytotoxicity induced by FL1-PNU. (**A**) Overview of GlyClick conjugation producing homogeneous DAR 2 PNU–conjugated ADCs (detailed in Materials and Methods). On the right is the chemical structure of the Fc-conjugated linker payload (shown as a red octagonal star on the left side of the mAb). (**B**) Dose-response curves showing the in vitro cytotoxic activity of PNU-FL1 ADC compared to NC ADC, aTNP-PNU as assessed by MTS assay. The percentage of cell viability relative to untreated control cells (*n* = 3, ±SD) is shown, while the fitted EC_50_ values are provided in Table 1. (**C**) Viability curves of various uPAR-positive cells following preincubation with the lysosomal protease inhibitor, (100 μM E64d), and LRP1-antagonist, (500 nM RAP), before a 72-hour exposure to FL1-PNU and NC ADC (*n* = 3, ±SD). All assays were performed as three independent replicates.

To conjugate FL1 to PNU, we used a chemoenzymatic glycoconjugation method (GlyClick, from Genovis) (Fig. 3A) (*25*, *26*). This technology enables the site-specific incorporation of the payload on the conserved N297-linked glycans in the fragment crystallizable (Fc) region of the antibody via click chemistry, yielding homogenous ADCs with a defined drug-to-antibody (DAR) ratio of 2.0 (Fig. 3A) (*25*, *26*). As FL1 efficiently traffics to the lysosomes, we opted for a valine-citrulline (VC)–based protease cleavable linker to allow for lysosomal cathepsin-mediated cleavage and release of the free drug inside the targeted cells (*27*). As a nontargeting control (NC), an isotype-matched mAb, directed against trinitrophenol, aTNP, was used and subjected to the same method of toxin conjugation. Successful payload coupling was confirmed for both ADCs, along with a DAR of 2, as revealed by SDS–polyacrylamide gel electrophoresis (SDS-PAGE) and ultraviolet-visible (UV-vis) analyses (fig. S5).

These PNU-mAb conjugates were tested in vitro by 5-day incubation with our PDAC cell panel (Fig. 3B). This experiment revealed a clear dose-dependent cytotoxicity of FL1-PNU with efficiency in the picomolar range (Table 1). While it was evident that higher uPAR expression levels correlated to lower median effective concentration (EC_50_) values, we noticed appreciable sensitivity even in cells with lower receptor counts, namely MIA PaCA-2 or BxPC-3. Reassuringly, for the uPAR-deficient KPC cells, there was no difference in the sensitivity profiles of FL1-PNU and the NC ADC, aTNP-PNU. These data demonstrated pronounced on-target cytotoxicity by FL1-PNU and defined a relatively broad specificity index.

**Table 1.** EC_50_ and specificity index of FL1 PNU relative to aTNP PNU on the eradication of PDAC cells. The numbers of uPAR molecules per cell (Fig. 1A) are shown for comparison. EC_50_ values were estimated from graphs shown in Fig. 3B using four-parameter logistic fitting on GraphPad Prism version 9. FL1-PNU displays a wide therapeutic window compared to aTNP PNU.

Cell lines	uPAR per cell	EC_50_ (nM)		
		FL1-PNU (a-uPAR)	aTNP-PNU (aTNP)	Fold difference (EC_50_ aTNP/EC_50_ FL1)
**MDA-MB-231**	1.3 × 10^6^	0.005	18	3600
**AsPC-1**	1.8 × 10^5^	0.08	4	50
**BxPC-3**	8.7 × 10^4^	0.05	12	240
**MIA PaCa-2**	1.4 × 10^4^	0.3	11	37
**KPC1**	2.4 × 10^5^	0.03	88	2933
**KPC1 uPAR** ^**KO**^	4.4 × 10^2^	17	36	2
**KPC2**	5.7 × 10^5^	0.02	18	900
**KPC2 uPAR** ^**KO**^	5.6 × 10^2^	12	18	1.5

To validate the mechanism of FL1-PNU internalization and lysosomal processing, the cell viability assays were conducted in the presence of LRP1 and lysosomal protease inhibitors. As shown in Fig. 3C, preincubation with E64d abrogated the FL1-PNU antitumor effect on the target cells. In contrast, the presence of RAP did not mitigate toxicity. These results demonstrated that FL1-PNU, like the parent mAb, traffics into the lysosome independently of LRP1 and requires active lysosomal processing of the cleavable linker to unleash its cytotoxicity. Flow cytometry and immunoblotting analyses of cell cycle and apoptosis in treated KPC2 and KPC2 uPAR^KO^ cells revealed that FL1-PNU on-target cytotoxicity involves poly(adenosine diphosphate–ribose) polymerase (PARP)–dependent apoptosis following cell cycle arrest in the S phase, typical of DNA-damaging payloads like PNU (fig. S6) (*28*, *29*). In summary, our in vitro data demonstrate that FL1-PNU efficiently delivers PNU into uPAR-expressing PDAC cancer cells, leading to potent cell killing.

### FL-1-PNU targets uPAR-positive immune and stromal cells and elicits bystander cytotoxicity in vitro

**Fig. 4.**
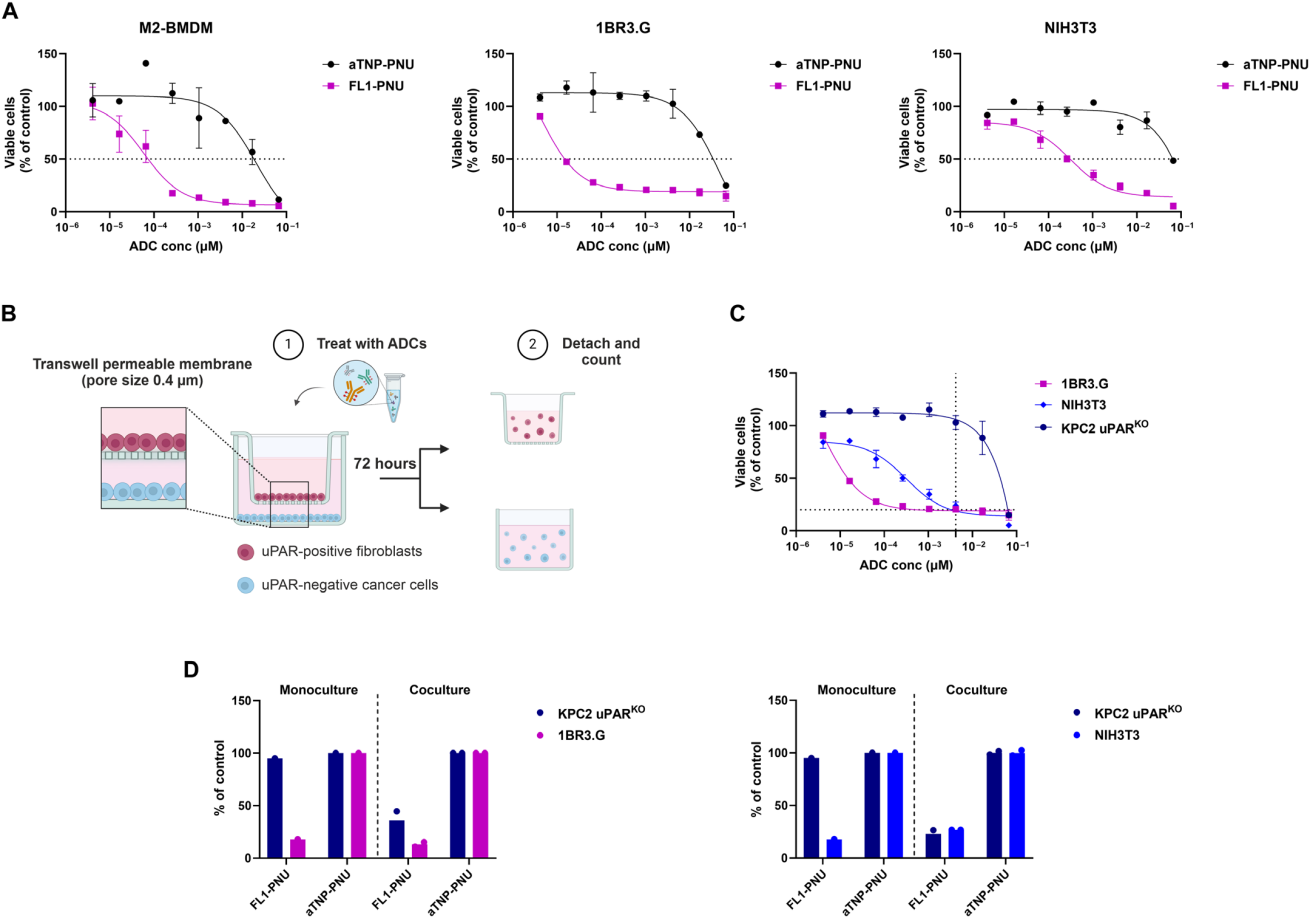
FL1-PNU targets uPAR-positive immune and stromal cells and induces bystander killing of uPAR-negative cancer cells. (**A**) Cell viability profiles of M2-polarized BMDM, human and murine fibroblast cell lines, 1BR3.G, and NIH3T3, as assessed by MTS assay following a 3-day exposure to FL1- and aTNP-PNU ADCs (*n* = 3, ± SD). (**B**) Scheme of established transwell coculture systems. uPAR^KO^ KPC2 cancer cells were seeded in six-well transwell plates, while uPAR-positive fibroblasts (1BR3.G or NIHT3T) were seeded in the upper transwell permeable inserts with a membrane pore size of 0.4 μm. FL1- and aTNP-PNU ADCs were added at 4 nM [EC_80_ for 1BR3.G and NIH3T3, (**C**)] to the upper chamber, and viable cells in both compartments were quantified after 72 hours of incubation. The percentage of cell viability relative to aTNP-PNU–treated cells (referred to as control) is represented (**D**) (*n* = 2).

Given the established negative impact of the dysplastic TME in PDAC drug resistance, simultaneous targeting of cancer, stromal, and immunosuppressive components may enhance the therapeutic response. Therefore, we investigated FL1-PNU’s activity on M2-differentiated bone marrow–derived macrophages (M2-BMDMs) and human and murine fibroblast cell lines, namely 1BR3.G and NIH3T3. These cells served as in vitro models of tumor-associated macrophages and activated CAFs, which dominate the PDAC TME and are known to up-regulate uPAR (*11*, *30*), compared to normal quiescent cells (fig. S7). Similar to the cancer cells (Fig. 3A), both M2-BMDM and fibroblasts exhibited a dose- and uPAR-expression–dependent response to FL1-PNU, compared to aTNP-PNU, following a 3-day exposure to the ADCs (Fig. 4A).

We next assessed whether this uPAR-mediated stromal/immune targeting by FL1-PNU could also promote the killing of uPAR-negative cancer cells by bystander cytotoxicity. For this purpose, we used dual-chamber Transwell dishes with uPAR-positive fibroblasts seeded in the upper permeable inserts and uPAR^KO^ KPC2 cancer cells in the bottom wells (Fig. 4B). In this system, any potential bystander factors can diffuse through the porous membrane to the bottom compartment. On the basis of the monoculture results, we selected a concentration of 4 nM FL1-PNU to achieve minimal targeting of the uPAR-null cells and maximum cytotoxicity of the uPAR-positive fibroblasts (EC_80_) (Fig. 4C). Both mono- and transwell cocultures were treated with the FL1- and aTNP-PNU ADCs, and the cell viability was assessed after 3 days of incubation. As expected, in the monoculture setting, direct FL1-PNU treatment markedly affected the viability of the uPAR-positive fibroblasts, but not of the uPAR^KO^ KPC2 cells, when compared to the aTNP-PNU ADC. However, in the coculture systems, we observed a notable reduction in both uPAR-positive and negative cell populations following exposure of the uPAR-positive fibroblasts to FL1-PNU (Fig. 4D). These results indicated that free PNU diffused in the media from the FL1-PNU–targeted uPAR-positive fibroblasts, resulting in the killing of the neighboring uPAR^KO^ cells, which are sensitive to PNU itself (fig. S4) but not to the uPAR-ADC. Combined, these data indicate that FL1-PNU can effectively target stromal and immune cells in a uPAR-dependent fashion. This leads to free drug release and bystander killing of cytotoxin-sensitive uPAR-negative tumor cells.

### FL1-PNU induces tumor regression in distinct mouse models of PDAC

We next assessed the ADC therapeutic performance in vivo both in xenograft and allograft mouse models of PDAC, taking advantage of the huPAR and muPAR cross-species reactivity of FL1. To establish a safe starting dose, we first conducted a preliminary tolerability study in naïve immunocompetent CD1 mice (fig. S8A). Two doses of 1 mg/kg administered intravenously once a week were well tolerated in CD1 mice with negligible weight loss and toxicity of vital organs as assessed by histology (fig. S8, B and C). In common with other ADCs (*31*–*33*), primary dose-related toxicity was predominantly detected in bone marrow/hematologic systems (fig. S8, C and D). Such toxicity was deemed negligible as these reactive changes are generally transient and reversible (*32*, *33*).

**Fig. 5.**
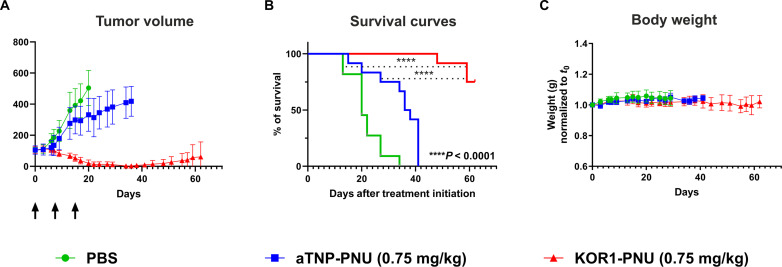
FL1- PNU suppresses established AsPC-1–derived subcutaneous xenografts. (**A**) Balb/c nude mice were engrafted with 2.5 million AsPC1 tumor cells by subcutaneous injection. When the mean tumor volume reached 50 to 120 mm^3^ (*n* = 11 to 12 mice per group), they were dosed three times with intravenous injections of vehicle or FL1-PNU or aTNP-PNU (0.75 mg/kg) (arrows). The recorded tumor growth curves throughout the study are displayed. (**B**) Kaplan-Meier curves displaying the extended overall survival of tumor-bearing mice after treatment with FL1-PNU as opposed to NC ADC– and vehicle-treated arms, respectively. The *P* values determined via the log-rank test are indicated. (**C**) Average mouse body weight (±SD) for each treatment group normalized to weight at treatment start demonstrates no significant difference between the groups over the observation period.

Building on this safety and tolerability profile, we performed the first treatment study in a xenograft model (Fig. 5). Immunodeficient Balb/c nude mice were subcutaneously engrafted with human AsPC1 cells. To further control for potential toxicity, we refined the ADC dosage regimen in Balb/c mice and lowered the concentration to 0.75 mg/kg of both FL1-PNU and aTNP-PNU. This dosage was overall well tolerated in naïve CD1 mice, where the acute-hematological effects we observed started reversing already 3 weeks after treatment (fig. S9). Tumor-bearing animals were administered with three cycles of ADCs or vehicle control and subsequently followed for seven more weeks from the last injection—a total of 62 days.

Already after the second dose, all FL1-PNU–treated mice (*n* = 12) showed pronounced tumor regression and complete tumor eradication was attained in 11 animals after the third dose at day 20 (Fig. 5A). In five mice, small palpable tumors reoccurred around day 48 but growth subsequently halted to yield a stable disease until the end of the observation period (fig. S10). The remaining six animals were cured, showing no sign of relapse over the entire follow-up. The observed antitumor effect translated to a highly significant long-term survival benefit, with nine mice (75%) surviving until the end point (Fig. 5B). In notable contrast to FL1-PNU–treated mice, both vehicle-treated (*n* = 11) and aTNP-PNU–treated (*n* = 12) groups showed rapid cancer outgrowth and reached humane end points for termination already after 20 and 37 days, respectively (Fig. 5B). Consistent with our in vitro observations, this result confirmed that uPAR-targeting by FL1-PNU results in a potent antitumor effect.

Notably, there were no changes in weight or abnormal behavior in any of the groups, further supporting the tolerability of FL1-PNU at this dosage (Fig. 5C). Furthermore, the treated animals presented with histologically normal bone marrow (fig. S11A), and normal hematological parameters, except for lower platelet counts observed in 2 of 12 mice (fig. S11B).

**Fig. 6.**
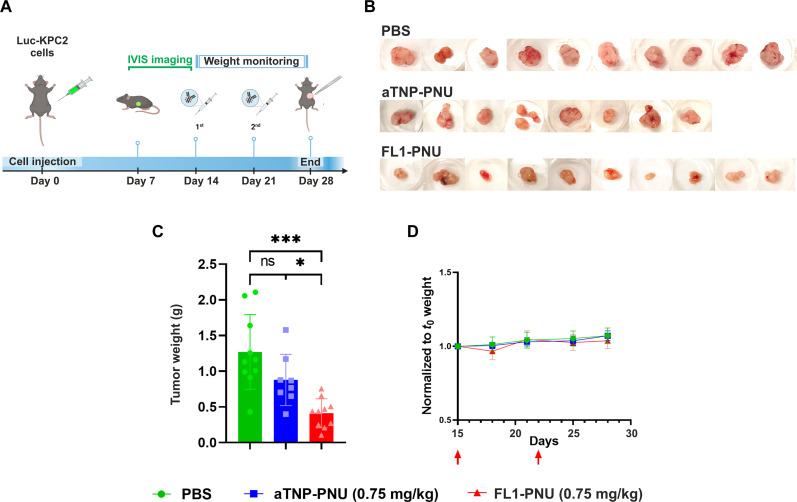
FL1-PNU impairs the growth of syngeneic KPC2-derived orthotopic allografts. (**A**) Study design and timeline. Female C567BL/6 mice (*n* = 8 to 10 mice per group) were orthotopically injected into the head of the pancreas with Luc-KPC2 cells (5 × 10^4^). The tumor implant was monitored by IVIS imaging. After 14 days, the mice were randomized and treated following the same weekly dosing schedule as in the xenograft study displayed in Fig. 5, for a total of two doses. (**B**) Pictures of harvested tumors at study termination on day 28. (**C**) Mean tumor weight (±SD) for each treatment group. (**D**) Mouse body weight was measured twice a week and expressed relative to initial weight at treatment start. The average (±SD) per treatment arm is shown. Statistical significance is indicated as follows: ns, non-significant; *P ≤ 0.05, ***P ≤ 0.001.

We next examined the therapeutic potential of FL1-PNU in an orthotopic PDAC model, encompassing a desmoplastic stroma, to assess the ADC effect in a physiologically relevant setting (*17*, *34*). To establish this model, murine KPC2 cells stably expressing Luciferase (Luc-KPC2) were implanted in the pancreas of syngeneic C57BL/6 mice (Fig. 6A). Our initial pilot studies showed that the use of Luc-KPC2 cells allows for real-time monitoring of tumor engraftment by IVIS imaging. However, the tumor size could not be accurately measured over time using this method because of the substantial tumor burden and deep location of orthotopic KPC2 tumors, which may hinder light penetration and limit luciferase substrate diffusion (*35*). As a result, we assessed treatment efficacy by comparing tumor weight at study termination.

When measurable tumor growth was detected, the mice (*n* = 8 to 10) were randomly assigned to the three treatment arms (Fig. 6A). Two doses of ADC were administered intravenously on days 14 and 21, respectively, and the study was terminated on day 28 because of the high tumor burden in the vehicle group. Also in this model, FL1-PNU induced a significant antitumor effect reflected by a 3- and 2.2-fold reduction of final tumor weights compared to the vehicle- and isotype-control ADC-treated groups, respectively (Fig. 6, B and C). Similarly, the ADC appeared well tolerated as no body weight changes or overt clinical signs of toxicity were noticed compared to the control arms (Fig. 6D).

### uPAR expression in tumors is preserved after FL1-PNU treatment

**Fig. 7.**
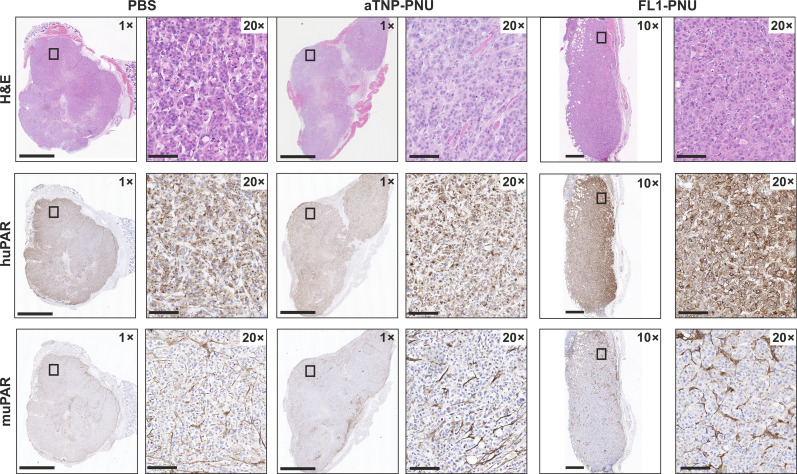
Histological assessment of control and relapsed AsPC-1-flank xenografts. Representative H&E stains and IHC analysis of uPAR expression in tumor sections from flank AsPC-1–xenografted mice shown in Fig. 5A. Despite the absence of observable tumors in most FL1-PNU–treated mice, a subset (5 of 12) developed slow-growing palpable tumors after treatment (>45 days), enabling detailed IHC examination. uPAR expression on human AsPC-1 cancer cells (stained for huPAR) and infiltrating murine fibroblasts in the stroma (stained for muPAR) is preserved after treatment with FL1-PNU, as seen in the control arms. Scale bars, (1×) 2.5 mm; (10×) 250 μm; (20×) 100 μm.

**Fig. 8.**
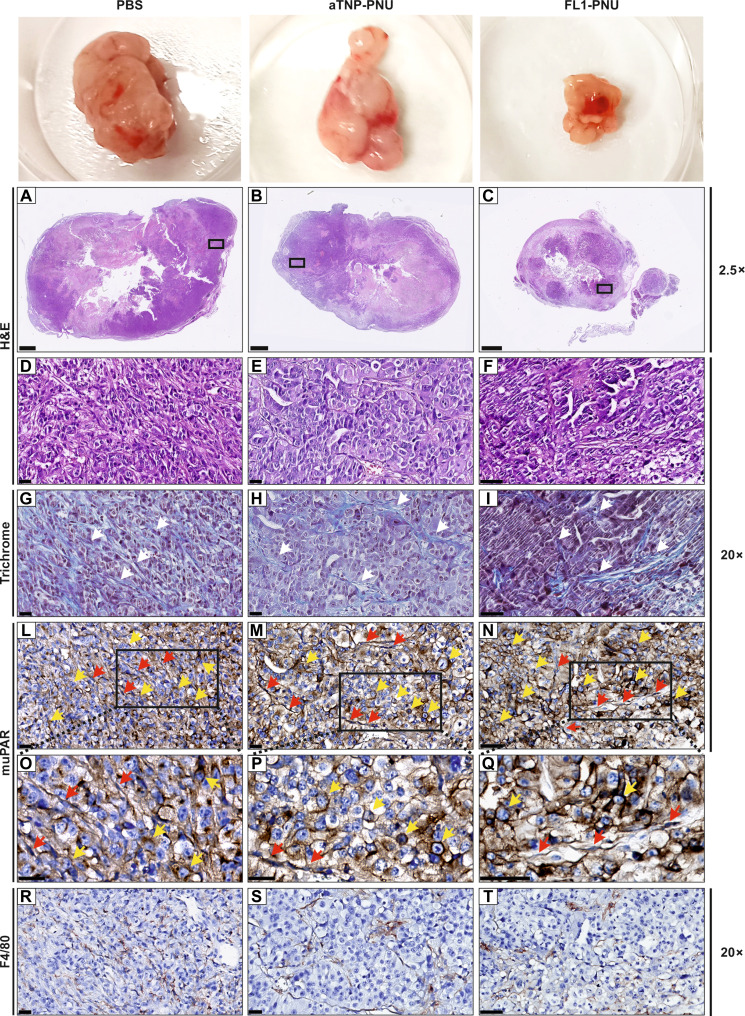
Histological assessment of KPC2-derived allografts. Examples of H&E (**A** to **F**), Trichrome (**G** to **I**), and IHC staining of muPAR (**L** to **Q**) and F4/80 (broad macrophage marker, **R** to **T**) in KPC2-derived syngeneic allografts, displayed in Fig. 6B. The black squares in (A) to (C) are shown in higher magnification in (D) to (Q). Collagen fibers, stained in blue in (G) to (I), are highlighted by white arrows, while red and yellow arrows in (L) to (N) indicate muPAR expression on stromal fibroblasts and cancer cells, respectively. Higher magnifications of the black box inserts in (L) to (N) are shown in (O) to (Q), where the spindle-shaped fibroblasts with flattened, oval nuclei can be distinguished from the round-ovoid cancer cells with large irregular nuclei and abundant cytoplasm. Brown patches in (R) to (T) indicate F4/80-positive stromal macrophages localized in matching uPAR-immunoreactive areas of the tumor sections. Scale bars, (2.5×), 1 mm; (20×) 100 μm.

Resected tumors from both xeno- and orthotopic engraftments (Figs. 5 and 6) were examined by hematoxylin and eosin (H&E) staining and immunohistochemistry (IHC) (Fig. 7). As expected, a significant reduction in the number of viable cells was detected in the FL1-PNU–treated tumors from both studies, relative to the two control arms, as reflected by their smaller tumor size. We did not detect any cancer cells in resected tissue from the xeno-engraftments of the cured mice, which mainly consisted of residual scar-like tissue, thus confirming histological remission (fig. S12). Nonetheless, resections of the few relapsed tumor sites revealed a pronounced uPAR expression in cancer cells as well as in the mouse-derived tumor-activated stroma, particularly fibroblasts (Fig. 7). This was indistinguishable from the pattern observed in the two other treatment groups. Staining of the orthotopic KPC2-allografts revealed that after the partial regression following FL1-PNU treatment, the remaining tumors likewise had persistent cancer and stromal uPAR expression (Fig. 8). In conclusion, these findings suggest that relapse, as well as partial regression, following FL1-PNU treatment, was the consequence of an incomplete eradication of uPAR-positive cells rather than an acquired resistance due to loss or down-regulation of the target Ag expression (see Discussion) (*36*).

### FL1-PNU reshapes the suppressive TME by promoting immunomodulation

A large body of in vivo evidence indicates that some ADCs can augment the efficacy of ITs, like ICIs, by activating antitumor immunity, for example, through the induction of tumor-infiltrating lymphocytes (*10*, *37*–*43*). This effect seems to be mediated by the payload’s ability to induce an immunogenic type of cell death (*10*). As the pronounced desmoplastic stroma is a recognized driver of the poorly immunogenic PDAC phenotype and resistance to ITs, we sought to investigate the effect of FL1-PNU ADC on the TME in the immunocompetent syngeneic KPC model.

**Fig. 9.**
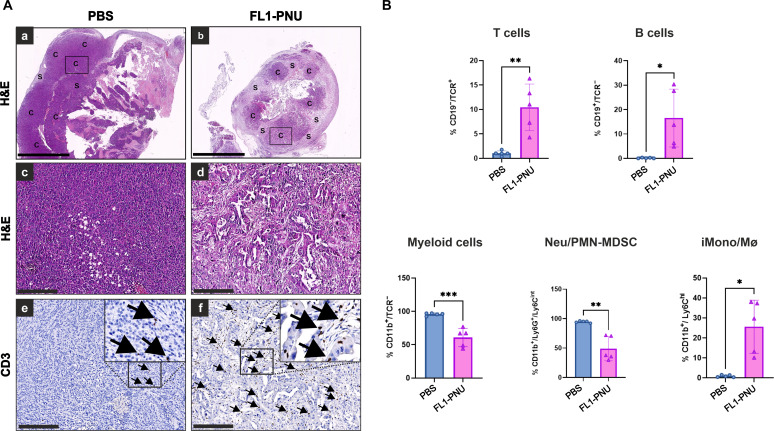
FL1-PNU antitumor effect remodels the suppressive immune landscape in syngeneic KPC-derived orthotopic tumors. (**A**) Representative IHC images of CD3 staining showing increased infiltrating T cells in FL1-PNU–treated tumors (f) compared to the sparse infiltrates in vehicle-treated tumors (e). The boxed area is shown at higher magnification to better appreciate the difference in the CD3-positive signal. A black insert in the corresponding H&E stains in (a) and (b) highlights the cancer (c) dense areas, presented in (c) to (e) and (d) to (f), respectively. S, stroma. Scale bars, [(a) and (b)] 2.5 mm; [(c) to (f)] 50 μm. (**B**) Immune profiling of main myeloid and lymphoid cell populations as quantified by flow cytometry (see graph subtitles and fig. S13 for gating strategy). *P ≤ 0.05; **P ≤ 0.01; ***P ≤ 0.001.

First, we qualitatively assessed the presence and distribution of T lymphocytes within the tumors by IHC CD3 staining. We observed a significant infiltration of T cells in both stromal and cancer areas of FL1-PNU–treated tumors, as opposed to the characteristic sparse infiltrates seen in the vehicle-treated ones (Fig. 9A).

To corroborate this finding and decipher the general ADC impact on the immune landscape, we performed immunophenotyping on a larger sample size, focusing on principal lymphoid and myeloid lineage markers (Fig. 9B). Strikingly, this analysis revealed a robust increase of both T and B lymphocytes in the FL1-PNU–treated samples, with these cells being almost absent in the phosphate-buffered saline (PBS) group. This result showed that FL1-PNU promoted adaptive cellular immunity.

When looking at the myeloid (CD11b^+^) cell compartment, we noticed that this population, which constituted nearly 90% of immune cells in the control samples, was almost halved in the FL1-PNU group. Similarly, we found a reduced number of the Ly6G^+^/Ly6C^lo/int^ cells, corresponding to neutrophils (neu)/polymorphonuclear myeloid–derived suppressor cells (PMN-MDSC), which dominated the myeloid compartment in the vehicle-treated samples. Furthermore, the Ly6C^hi^ cells identifying monocytes or macrophages with the classical inflammatory phenotype (iMono/Mø), which were lacking in vehicle-treated tumors, were significantly expanded in the ADC-treated group. These findings indicated that the potent antitumor activity by FL1-PNU contributes to reshaping the immunosuppressive TME by promoting infiltration of adaptive and innate immune players and reduction of myeloid suppressive cells.

## DISCUSSION

The present study provides the first preclinical validation of uPAR as a promising target for ADC therapy in PDAC. Our recently developed anti-uPAR ADC, FL1-PNU, demonstrated robust and specific antitumor responses in aggressive PDAC models, in vitro and in vivo, both in subcutaneous xenograft and orthotopic allograft models, with a tolerable safety profile. In the orthotopic model, the evident tumor reduction induced by FL1-PNU was accompanied by TME remodeling and immunomodulation by enhancing lymphocyte and inflammatory cell infiltration while decreasing suppressive myeloid cells.

ADCs present an attractive therapeutic approach for treating pancreatic cancer; however, their clinical translation has not yet been successful compared to other solid cancers (*1*, *7*, *8*). Factors including the hostile and immunosuppressive PDAC TME and the selection of suitable candidate targets and/or antibodies are among the clinical challenges of investigational ADCs in patients with PDAC (*4*, *5*). Most explored targets have generally shown insufficient tumor specificity and/or internalizing capacity (*1*, *8*). On the other hand, the mAb components were often chosen for their antagonistic rather than uptake properties, which is a determinant factor for the therapeutic activity of internalizing ADCs (*7*, *8*, *44*).

Owing to its marked overexpression in PDAC tumor tissue and concomitant stromal abundance, uPAR represents an attractive target in this malignancy (*11*, *13*). While endogenous internalization of uPAR has been characterized, evidence on receptor-mediated uptake of targeted therapies, including mAb-based reagents, is not extensive (*11*). Our in vitro data clearly demonstrate that uPAR has efficient endocytic and lysosomal trafficking activity, rendering it a viable ADC target in PDAC. We constructed our uPAR-specific ADCs to incorporate a highly internalizing mAb candidate, FL1, selected from a panel of high-affinity anti-uPAR mAbs (Fig. 1). We showed uPAR-specific binding and uptake of FL1 in various differentially expressing PDAC cell lines and, importantly, found that even cells with relatively low receptor numbers were capable of substantial internalization of the antibody (Fig. 2).

FL1 shows cross-species reactivity and recognizes both human and mouse uPAR (Fig. 2), thus allowing both studies in xenografted and syngeneic tumors and an initial assessment of ADC on-target toxicity (*11*, *45*). Furthermore, this feature allows for assessing ADC efficacy in more physiologically relevant syngeneic orthotopic models (*34*). In stroma-rich cancers like PDAC, this model type enables an initial impression of the intratumoral payload delivery as well as the ADC impact on the TME. This is especially relevant when the target is also present on stromal cells, as for uPAR (*9*, *12*, *46*).

Only one example of a uPAR-targeted ADC has been reported. This ADC, a humanized uPAR-antagonist mAb, 2G10, conjugated with the tubulin inhibitor MMAE, has demonstrated preclinical efficacy in a xenograft model of TNBC (*47*). However, the use of an antibody exclusively directed against huPAR precluded a proper evaluation of toxicity or stromal targeting effects, which may influence the treatment efficacy. In addition, the therapeutic window was not defined because of the lack of a nonbinding ADC control (*47*).

As the ADC field continuously evolves, efforts are directed to diversify the payload arsenal to address resistant and advanced-stage cancers. So far, PDAC has been found refractory to ADCs based on canonical tubulin-inhibiting payloads (*1*, *8*). Given these findings, we opted for a clinical-stage anthracycline derivative, PNU-159682. This payload has lately raised awareness in the ADC landscape due to its superior potency, unique DNA-targeting MOA, and ability to overcome typical drug-efflux resistance mechanisms compared to anti-tubulin agents (*24*, *28*, *29*, *31*, *37*, *39*, *48*–*52*). Of the two PNU-based ADCs in clinical trials, SOT102, which targets CLDN18.2, has demonstrated a large therapeutic window even in low Ag-expressing tumors, including pancreatic cancer (*24*, *53*). Therefore, potent payloads may overcome the requisite threshold receptor density for efficacy, which is beneficial when targeting an Ag with a heterogenous expression like CLDN18.2 or uPAR. However, targets with efficient internalization or concurrent cancer and stromal expression, such as uPAR, might allow for more versatile payload selection and thus fine-tuning of the therapeutic window based on the target indication or patient population.

To incorporate such cytotoxic payloads, site-specific conjugation strategies are preferable over random coupling methods to obtain homogenous and stable ADCs with a defined DAR (*24*, *54*). By using a recently established glycoconjugation technology, GlyClick, we successfully obtained DAR 2 site–specific ADCs bearing a protease-cleavable linker (VC-PAB) (Fig. 3). This linker structure has been used successfully in several ADCs, including approved benchmark ADCs, like Adcetris (*6*, *7*, *55*). Another advantage of this technology is the ablation or reduction of Fc-mediated off-target toxicity or effector functions, following N297-linked glycan removal, which further improves ADC pharmacokinetics and tolerability (*7*, *54*, *56*).

FL1-PNU showed potent cytotoxicity and a wide specificity window in all uPAR-positive cells compared to the uPAR^KO^ ones and irrelevant NC ADC (Fig. 3). This effect was exclusively mediated by the targeted delivery of the payload, as unmodified FL1 did not affect any of the cell lines’ growth. Yet, our data also indicate that the product of uPAR expression and efficient endocytosis likely determines FL1-PNU efficiency; the latter factor may eventually compensate for lower uPAR receptor counts on the surface of some cell lines, as found for MIA PaCa-2 or BxPC-3 cells, and similarly found for HER2 or CD22 (*44*, *57*). Although preliminary, this finding may eventually support the applicability of the anti-uPAR ADC in low-uPAR–expressing patients, similar to the case of trastuzumab deruxtecan (T-Dxd, Enhertu), which was recently approved for patients with low-HER2 expression (*58*).

In addition to PDAC cells, FL1-PNU also showed strong activity against in vitro models of uPAR-positive tumor-associated macrophages and CAFs (Fig. 4). Direct targeting of these cells may help to alleviate the fibrotic and immunosuppressive barriers and impair the tumor-stroma cross-talks driving PDAC progression and metastasis (*1*, *4*, *30*). Furthermore, the ability of uPAR-positive cells to mediate a bystander effect after targeting by FL1-PNU, as exemplified by our transwell coculture experiments, may further amplify the antitumor response by facilitating intratumoral drug distribution within tumors with heterogeneous uPAR expression, like PDAC (*11*, *13*, *59*).

In vivo, FL1-PNU was well tolerated at the optimized dosage of 0.75 mg/kg under the applied treatment regimens. Notably, vital organs, namely the heart and lungs, along with the liver and kidney, which typically exhibit low levels of endogenous uPAR (*60*), were not apparently affected (fig. S9). In addition, compared to the dose-escalation study in CD1 mice, we did not observe evidence of payload-induced myelotoxicity in the xenograft model, except for two cases of thrombocytopenia (2 of 12 mice) (*32*, *33*). Although we cannot exclude potential on-target effects on blood cell populations like monocytes and neutrophils, which display the highest baseline uPAR levels (*61*), our findings demonstrate that these effects are typically dose dependent and reversible upon treatment withdrawal (figs. S9 and S11B). Furthermore, the absence of uPAR on progenitor blood cells (*61*) eliminates the risk of irreversible myelosuppression that could compromise treatment. Nonetheless, further toxicological investigations are necessary to fully evaluate the safety profile of our ADC.

In both mouse models tested, FL1-PNU had clear antitumor effects at the low administered concentration (Figs. 5 and 6), with complete remission achieved in the xenograft model (6 of 12 mice). Congruent with other reports, we also noticed a mild effect on tumor growth by the NC ADC (*1*, *7*, *51*, *62*, *63*). This phenomenon may likely result from a combined enhanced permeability and retention effect in the tumors and nonspecific proteolytic processing of the ADC in the extracellular space (*6*, *7*, *9*). The reduced dosage schedule and shorter study time frame in the orthotopic model, enforced by the rapid tumor growth in vehicle-treated mice, may likely explain the absence of complete responders and the narrower therapeutic window between the NC and FL1-ADC compared to the xenograft study. Nevertheless, despite the high tumor burden and dense stromal barrier seen in this model, the target-specific therapeutic effect was still statistically significant.

The pronounced uPAR expression observed by IHC in residual or recurrent tumors in both in vivo models after treatment with FL1-PNU (Figs. 7 and 8) points to a lack of resistance resulting from target down-regulation or loss (*36*). This may enable a prolonged treatment regimen or even retreatment with the current ADC. Extending the treatment follow-up will also help further elucidate the safety profile of the ADC or reveal resistance mechanisms that have not emerged during these studies.

When looking into the immune cell compartment of the syngeneic KPC-derived model, we noticed a significant increase in both adaptive and innate immune players in the FL1-PNU–treated tumors suggesting an immunomodulatory effect elicited by the ADC (Fig. 9). Similar findings have been reported with other ADCs, including conjugates with anthracycline payloads (*10*, *37*–*43*). As expected, the control samples were dominated by suppressive myeloid cells, particularly neutrophils, and PMN-MDSC (*4*). Notably, these cells were significantly decreased in the FL1-PNU–treated tumors. This effect may derive from a direct receptor targeting on these cells by the ADC, as supported by our in vitro data and likewise observed by others (*11*). However, we do not exclude that the altered uPAR-dependent and independent cancer-stroma cross-talk following cancer cell eradication may impair myeloid cell recruitment while favoring T cell infiltration. Similar immunomodulatory effects following uPAR targeting via an mAb and derived cytotoxic CAR-T cells have recently been described (*64*). Both uPAR-targeting strategies were found to significantly inhibit tumor growth and activate the immune system in humanized cell-derived and patient-derived xenograft models of gastric cancer. When combined with anti–PD-1 blockade, the combination regimens showed additive antitumor and immunostimulatory activities. These findings align with our data and may suggest a relevant role of the uPAR-axis in the TME dynamics and tumor progression in these cancers. While comprehensive immune profiling is needed to confirm and dissect the proposed mechanism, it would be interesting to verify whether treatment with FL1-PNU likewise synergizes with ICIs and promotes immunological memory (*10*, *37*–*43*). In this perspective, targeting the uPAR-positive myeloid-suppressor cells may facilitate an activated antitumoral immunity by relieving immune tolerance. While evidence of immunostimulatory activity by ADCs is still limited in PDAC (*1*), such an approach may provide a promising therapeutic avenue for treating these patients.

To conclude, our preclinical findings are well aligned for a future clinical translation of FL1-PNU as a uPAR-targeting ADC, either alone or in combination with other ITs, like ICI. Studies in mouse models with negative or heterogenous uPAR expression, like patient-derived xenograft, will help further decipher the ADC potency in vivo and dissect the uPAR-driven stromal and immune cell contribution to the therapeutic effect. The availability of clinical-stage uPAR-imaging agents, which have proven successful for noninvasive positron emission tomography– or magnetic resonance imaging–based evaluation of prostate, bladder, and breast cancers (*27*, *65*), may provide a reliable diagnostic companion for patient screening and therapeutic follow-up and lay the foundation for a precision medicine approach for PDAC and, plausibly other uPAR-positive cancer indications, with an unmet need.

## MATERIALS AND METHODS

### Cell lines

Hybridomas producing the anti-uPAR antibodies presented in this work and the isotype-matched NC mAb directed against trinitrophenol were established in-house as described in (*60*, *66*–*71*). The cell lines investigated were obtained from the following sources: AsPC-1 [ATCC American Type Culture Collection (ATCC) #CRL-1682], BxPC-3 (ATCC #CRL-1687), MIA PaCa-2 (ATCC #CRM-CRL-1420), MDA-MB-231 (TNBC, ATCC #CRM-HTB-26), U937 (ATCC #CRL1593.2), NIH3T3 (ATCC #1658), and 1BR3.6 (ECACC, #90020507). The parental KPC cancer cells were individually generated from primary tumors of KPC (*Kras^G12D/+^*, *p53^R172H/+^*, *Elas^CreER/+^*) mice, as detailed in (*17*). The corresponding uPAR^KO^ clones were derived using a CRISPR RNA–guided Fokl nuclease strategy to introduce INDELs in both PLAUR gene’s alleles (*17*). The M2-polarized macrophages were derived from BMDMs, as described in fig. S7 and detailed in (*72*). Cell lines were maintained in a 37°C, 5% CO_2_ incubator in supplier-recommended media: hybridomas in RPMI 1640–GlutaMAX supplemented (Thermo Fisher Scientific, #61870036) with 10% Ultra-Low IgG fetal bovine serum, 1% antibiotic-antimycotic (Thermo Fisher Scientific, #15240096), 1% sodium pyruvate (Thermo Fisher Scientific, #11360070), and 0.1% 2-mercaptoethanol (Thermo Fisher Scientific, #21985023); U937, AsPC-1, BxPC-3, and all KPC cells in RPMI 1640–GlutaMAX; MDA-MB-231 and MIA PaCa-2 in Dulbecco’s modified Eagle’s medium (Thermo Fisher Scientific, #11965092). All media were supplemented with 10% fetal bovine serum and 1% penicillin-streptomycin; in addition, 2.5% horse serum was included in MIA PaCa-2 media formulation. All cell lines were genetically authenticated by the ATCC and tested negative for pathogens, including mycoplasma, by IDEXX BioAnalytics.

### Antibodies and proteins

All hybridoma-produced a-uPAR- and aTNP-mAbs were produced as outlined in (*60*, *66*–*71*). The rabbit a-huPAR and a-muPAR (P47) pAbs used in the WB and IHC analyses have been previously described in (*60*). Antibodies purchased from commercial sources are thereafter indicated. Recombinant soluble muPAR (smuPAR) and huPAR (I-III) (shuPAR) were produced in Drosophila Schneider S2, as detailed in (*45*, *68*, *71*, *73*–*75*).

### Fluorescence and radioactive labeling

A647-labeled mAbs were generated using a mouse IgG1 labeling kit (Thermo Fisher Scientific, #A20186), by the manufacturer’s instructions. Briefly, mAbs at 1 mg/ml in sodium bicarbonate buffer (pH 8.3) were incubated with a fivefold molar excess of A647 reactive dye (at 10 mg/ml in dimethyl sulfoxide) for 1 hour at room temperature (RT) in the dark, under gentle agitation, and then purified by gel filtration on PD-10 desalting columns. The A647 conjugates were characterized for their degree of labeling (DOL) by absorbance measurements, following the vendor protocol. The DOL ranged from 1.8 to 3.6.

For live-cell imaging, FL1 was labeled with an isotype-matched antibody-binding fragment (Fab) coupled to a pH-sensitive red fluorescent probe from Incucyte (FabFluor-pH Red, Sartorius, #4737), following the manufacturer’s protocol. Briefly, the test antibody at 10 μg/ml (two times the final required assay concentration) was mixed with the labeling reagent at a 1:3 molar ratio in complete growth media, for 1 hour at 37°C in the dark and then added directly to preplated cells, as specified in the “Live-cell imaging” section. ^125^I labeling of the candidate mAb FL1 was carried out as described in (*76*), followed by reducing SDS-PAGE analysis and visualization on a Phosphor Imager (Fuji Fla. 3000).

### Western blot

Cells were lysed in radioimmunoprecipitation assay buffer (Thermo Fisher Scientific, #8990) supplemented with protease inhibitor cocktail III (1:200; Sigma-Aldrich, #535140) for 20 min on ice, followed by high-speed centrifugation (20,000*g* for 15 to 20 min at 4°C). Total protein content was measured using the Pierce bicinchoninic acid kit (Thermo Fisher Scientific, #23225). Twenty to thirty-five micrograms of total protein per sample was resuspended in NuPAGE sample buffer (Invitrogen, #NP0008), boiled, and electrophoresed on NuPAGE 4 to 12% gradient bis-tris gels (Invitrogen) for 1 hour at 200 V using 1× MOPS SDS as running buffer (Invitrogen, #NP0001-02). Purified recombinant smuPAR (20 ng) and shuPAR (10 ng) were included as positive controls in their respective gels. The samples were subsequently transferred onto polyvinylidene difluoride membranes (Invitrogen, #IB401001) using the iBlot Dry Blotting system (Invitrogen) for 7 min, followed by total protein detection using the Revert 700 stain kit per vendor’s instructions (LI-COR, #926-11011). Afterward, the membranes were blocked in 10% skim milk powder (Sigma-Aldrich, #70166-500G) in PBS + 0.1% Tween20 (pH 7.4) for 1 hour at RT. Immunoblotting was then performed by incubation with indicated primary antibodies [diluted at 1 to 2 μg/ml in PBS with 0.1% Tween 20 (pH 7.4)] at 4°C followed by washing in PBS with 0.1% Tween 20 (pH 7.4) and detection with a goat anti-rabbit IgG (H + L) highly cross-adsorbed, Alexa Fluor Plus 800 secondary antibody [diluted 1:8000 for the human cell blot and 1:10,000 for the mouse cell blot in PBS with 0.1% Tween 20 (pH 7.4) Invitrogen, #A32735]. Incubation was conducted at RT for 45 min in the dark. Blots were imaged at 700 and 800 nm and visualized using the Odyssey application software Image Studio (version 5.2).

For PARP determination, membranes were probed with rabbit anti-PARP primary mAb (Cell Signaling Technology, #9532S; 1:1000) and IRDye 800CW goat-anti-rabbit IgG secondary antibody (Li-COR, #926-3221; 1:10,000). Cytosolic HSP90 levels were used as a loading control and detected using HSP90 alpha/beta primary mAb (F-8, Santa Cruz Biotechnology, #sc-13119) and IRDye 680RD goat anti-mouse IgG secondary antibody. The membranes were scanned at 800 and 680 nm on the ChemiDoc MP imaging system (Bio-Rad).

### Quantification of cell surface uPAR by flow cytometry

Surface uPAR density was quantified using QSC calibration microspheres (Bangs Laboratories, #816), as per the manufacturer’s protocol. Briefly, cells (at 80 to 90% confluence) were harvested using 5 mM EDTA in PBS and rinsed twice in ice-cold fluorescence-activated cell sorting (FACS) buffer [0.1% bovine serum albumin (BSA), in 1× PBS] through suspension-spin cycles. A total of 0.5 × 10^6^ cells/100 μl per cell line, along with the five prepared QSC standard bead populations, were incubated with A647-labeled FL1 at a predetermined saturating concentration on ice in the dark for 1 hour. After staining, the samples were washed twice and resuspended in FACS buffer supplemented with 1 μg/ml with 7-AAD (Invitrogen, #1890506) for cell death exclusion. The samples were acquired on a BD LSR Fortessa 20X (BD Biosciences). A total of 1000 and 10,000 events of the desired gated population were collected per each standard bead population and cell line, respectively. Data were analyzed using the FlowJo v10.8.1 software. The receptor number was determined by interpolating the cell samples mean fluorescence intensity (MFI) values (corrected for the autofluorescence of corresponding unstained samples) to a standard curve associating every calibration bead’s MFI to its preassigned antibody-binding capacity, using the lot-specific QuickCal v.2.3. template.

### Internalization assays

Intracellular uptake of the examined anti-uPAR mAbs was evaluated through different approaches.

#### Fluorescence- and radioactive-based internalization assays

Cells, seeded in triplicates in 24-well plates (10^5^ per well), were incubated with A647-labeled a-uPAR mAbs (5 μg/ml) for the indicated time points (4 and 20 hours) at 37°C to allow endocytosis. When indicated, A647-IgG1 was included as a nonbinding control (Invitrogen, #MG121). After incubation, the cells were collected by centrifugation, resuspended in FACS buffer, and analyzed by flow cytometry. When specified, the removal of surface-bound mAbs was performed by a short incubation with 0.25% trypsin-EDTA supplemented with proteinase K (50 μg/ml) before analysis. In the screening assay of the panel of A647-mAbs in U937 cells (Fig. 1), parallel cell samples were incubated at 4°C and used as “non-internalization” controls. After incubation, the cells were processed as per above, except for the protease treatment step.

The radioactive-based internalization assay was carried out according to a similar procedure, as detailed in (*76*). The amount of internalized radio-conjugate was measured using a γ-counter (Wizard^2^ Gamma Counter, PerkinElmer/Revvity) and represented as counts per minute.

### Confocal microscopy

Microscopy-based assessment of A647-FL1 uptake was performed in suspension U937 cells and adherent KPC cells. Triplicate samples were seeded at the density of 5000 cells per well in 24-well plates, with adherent cells plated on coverslips. After 4 hours of incubation at the indicated temperatures, unbound mAbs were removed by three washes with ice-cold 1× PBS. When indicated, surface staining was performed on live cells following 15 min of incubation at RT with A488-WGA (1 μg/ml in PBS, Invitrogen, #W11261), followed by three additional washing steps. U937 cells were cytocentrifuged at 500*g* for 5 min at 4°C onto microscope glass slides (Superfrost ultra plus, Thermo Fisher Scientific) using a Hettich Cyto-System and one-funnel chambers (Simport, #M966-4). The cells were then fixed in 4% paraformaldehyde (PFA) for 15 min at RT. The slides were mounted using ProLong Gold Antifade Mountant with 4′,6-diamidino-2-phenylindole (DAPI) as nuclear counterstain (Thermo Fisher Scientific, #P36941) and left to dry overnight. Image acquisition was performed on a Zeiss LSM 800 or LSM900 confocal microscope using a 40× oil objective. To investigate the effect of LRP1 receptor and lysosomal protease inhibition on receptor-mediated internalization, the cells were preincubated with 500 nM of murine or human recombinant RAP (Abcam, #ab201883 and #ab123156, respectively) and 100 μM of E64d protease inhibitor (Sigma-Aldrich, #330005) for 1 hour at 37°C before the assay.

### Live-cell imaging

Cells were plated overnight at optimal densities in triplicate wells of flat-bottom tissue culture–treated 96-well plates (Costar, #3595), before incubation with Incucyte Red Fabfluor pH–labeled-FL1 or labeling dye alone (5 μg/ml). Untreated control cells were included to correct for autofluorescence. The plates were imaged every 15 min for up to 24 hours in an Incucyte S3 live-cell analysis system (Sartorius) using a 10× objective. The acquired images were quantified for the related phase confluence (a measure of the total cell area) and red fluorescence signal (index of internalized antibody), via the Basic analyzer module of the Incucyte software. Background subtraction was performed using the “Top-Hat” function. The time course of internalization was represented by plotting the average integrated fluorescence intensity per cell line against time, while total uptake was calculated by normalizing to the corresponding phase confluence (%) to correct for differences in cell proliferation.

### ADCs production and characterization

Site-specific PNU-ADCs were generated by glycan- and click chemistry–based GlyClick conjugation with the click-reactive linker-toxin DBCO-PEG4-VC-PAB-DMEA-PNU-159682 (Levena, #SET0313). The GlyClick Azide Activation kit (Genovis, #L1-AZ1-200) was used to produce label-ready azide-modified mAbs, according to the manufacturer’s instructions. Briefly, antibodies in tris-buffered saline (TBS) were first deglycosylated by enzymatic hydrolysis of the conserved Fc N297–linked glycans to the innermost *N*-acetylglucosamine (GlcNAc) moiety using immobilized GlycINATOR. azide-containing UDP-GalNAz was then linked to the exposed inner GlcNAc using the GalT enzyme (Y289L), generating azide-activated mAbs. After overnight incubation at 30°C, these were purified by the removal of excess UDP-GalNAz on a 2 ml of desalting spin column (40 K MWCO; Thermo Fisher Scientific, #87768) and run on a reducing SDS-PAGE gel to confirm Az-modification before conjugation. The coupling reaction was then conducted in a 3:1 ratio (v/v) of Az-mAb (in TBS) to linker-payload (dissolved in 80% propylene glycol (Sigma-Aldrich, #P4347), 20% *N*,*N*′-dimethylformamide [Sigma-Aldrich, #DX1730-6) (v/v)]. The linker-payload was added in a 15× molar excess to Az-modified mAbs. Following overnight incubation at 25°C, the coupling reaction was assessed by SDS-PAGE analysis, and the conjugates were subsequently purified as per above. The concentration, purity, and DAR of the ADC products were further examined via UV-vis spectroscopy on a QIAxpert instrument, as detailed in fig. S5. The obtained Az-modified mAbs and ADCs were stored at 4°C in the dark.

#### Cell viability assay

Cells, plated in triplicates at 3 × 10^3^ cells per well in 96-well plates, were dosed with eight series of fourfold serially diluted PNU conjugates, unconjugated FL1, and free toxin (Creative Biolabs, #ADC-P-025) in 100 μl of complete growth media. Control cells were incubated with PBS-supplemented media. Cell viability was measured after 3 or 5 days of incubation, using CellTiter 96 Aqueous one-solution cell-proliferation assay reagent (MTS, Promega, #G358), as per the vendor’s recommendations. Plate absorbance was then measured at 490 and 650 nm (for background subtraction) using a Spectra Max Plus plate reader (Molecular Devices). Data were plotted as a function of drug concentration (nanomolar) in GraphPad Prism 9 and fitted to a four-parameter nonlinear regression function, from which EC_50_ values were derived.

#### Transwell coculture assay

The ADC bystander activity was assessed by a coculture of uPAR-positive fibroblasts, human 1BR3.G, and murine NIH3T3 cells, respectively, and uPAR^KO^ KPC2 cancer cells in dual-chamber transwell-clear dishes. A total of 7.5 × 10^4^ uPAR^KO^ KPC2 were plated in the bottom chamber of the six-well cell culture plates, while 1.5 × 10^5^ fibroblasts were seeded in the upper transwell inserts (Corning, #3450), with microscopically transparent polyester membranes with a pore size 0.4 μm, designed for drug transport studies. At 24 hours after seeding, FL1-PNU or aTNP-PNU ADCs (at 4 nM) were added to the upper inserts. Following 72 hours of incubation, the cells in both chambers were detached and viability was measured using a NucleoCounter NC-3000 (ChemoMetec) after staining for live/dead cells with Solution-13 AO-DAPI stain (ChemoMetec, #910-3013).

### Cell cycle and apoptosis analysis

KPC2 and relative uPAR^KO^ cells were seeded in six-well culture plates at optimal defined densities and treated with the indicated concentrations of FL1-PNU. After the specified time points, the cells were harvested by trypsinization and washed twice with ice-cold PBS. For the cell cycle analysis, the cells were fixed with ice-cold 70% ethanol overnight at –20°C, followed by DNA staining with propidium iodide (PI)/ribonuclease staining buffer (BD Biosciences, #550825). The apoptosis assessment was performed using annexin-V–fluorescein isothiocyanate apoptosis detection kit I (BD Pharmingen, #556547) following the vendor’s protocol. After washing, the cells were resuspended in 100 μl of annexin V binding buffer at 10^6^/ml and stained with 4 and 3 μl of annexin V and PI, respectively. In both assays, staining was conducted for 15 min at RT in the dark for 30 min before flow cytometry analysis on the Invitrogen Attune NxT. The percentage of cells in the four cell cycle phases, and apoptotic stages, were determined using the FlowJo v10.8.1 software.

### In vivo efficacy studies in xenograft and orthotopic PDAC models

All studies received legal approval from The Danish Veterinary and Food Administration and the University of North Carolina Animal Care and Use Committee under license number 2020-15-0201-00533 (L.H.E.) and IACUC: 22-164.0 (M.J.F.), respectively. All reagents and cell lines used were screened and tested negative for the presence of endotoxins, murine, and human viruses, bacteria, mycoplasma, and fungi (IDEXX BioAnalytics). The animals received standard care, and experiments were performed according to the principles of the 3Rs (refine, reduce, and replace).

The treatment studies in AsPC-1 cell line–derived subcutaneous xenografts were performed in 7- to 10-week-old female BALB/cAnNRj-Foxn1 nu/nu mice (JANVIER LABS). These were injected into the upper right flanks with sterile PBS (2.5 × 10^6^ cells/100 μl) per mouse. When tumors reached 80 to 150 mm^3^, the animals were block-randomized into treatment groups of 11 to 12 according to mean tumor size and body weight and then administered with a weekly tail intravenous injection of test articles or vehicle control (0.75 mg/ml) for over three consecutive weeks. Tumor volume (mm^3^ = length × width^2^/2) and body weight were recorded two to three times weekly, and the mice were closely monitored for any signs of distress or abnormal behavior. Euthanasia was performed at predetermined endpoint criteria, respectively, tumor size exceeding 1000 mm^3^ or 12 mm in diameter, tumor ulcers more than 8 mm in length, ~15 to 20% weight loss, or when half of the mice of a treatment arm were terminated.

Syngeneic graft studies were performed using 8-week-old wild-type C57BL/6 mice (the Jackson Laboratories) orthotopically injected into the pancreas with Luc-KPC2 cells (5 × 10^4^) in 30 μl of sterile PBS, following an established surgical procedure. Two weeks after recovery, tumor engraftment was qualitatively assessed using the IVIS Spectrum Imaging System (PerkinElmer), following intraperitoneal injection of d-luciferin (PerkinElmer, #122799). Mice with no detectable luciferase activity were excluded from the study (2 of 30). The remaining animals (28 of 30) were randomized into the three treatment arms (*n* = 8 to 10) with equal average luciferase activity, dosed twice as described above, and finally euthanized at day 28 from the tumor implant. The treatment efficacy was evaluated by comparing tumor weight at termination.

In both studies, the mice were euthanized following Zoletil or Ketamine/Xylazine/ Acepromazine anesthesia. Tumors and specified organs were harvested after animal perfusion by intracardial injection of 10 ml of PBS and, when indicated, weighed, and finally prepared for histological examination or immune profiling, as detailed in the next sections. Blood was collected and analyzed with the Element HT5 Hematology Analyzer (Heska). All experiments were performed under a double-blinded setup with people responsible for injecting mice and assessing tumor volume being blinded from the identity of the treatment arms.

### Harvest and processing of mouse tissue for histological examination

Excised tumors and indicated organs were transferred to a 4% PFA solution for histological processing. After 24 hours of incubation at 4°C, the samples were moved to 70% ethanol. Bones were decalcified in a 10% water solution of EDTA (pH 7.4) by microwave heating at 50°C and 600 W for 20 min, followed by 2 hours at the same temperature and 300 W. To ensure complete decalcification, the femurs were stored in fresh 10% EDTA for 3 days at 4°C. All samples were dehydrated with increasing concentrations of ethanol and subsequently embedded in paraffin. The embedded tissue samples were sectioned at 3.5 μm on an automatic microtome (Thermo Fisher Scientific, #HM355S) and mounted on glass slides.

### Histology and IHC

Tissue sections were deparaffinized [using tissue clear (Sakura finetek, #1466) and decreasing ethanol concentrations], rinsed in tap water, and subjected to the indicated stainings as follows: H&E stain was performed on an automated slide stainer (Gemini), with hematoxylin (HARRIS HTX solution, HistoLab, #01820) and eosin (eosin solution 0.2%, HistoLab, #01650) for 5 min each with rinsing in between. Samples were finally dehydrated with increasing concentrations of ethanol, dried for 10 min, and mounted with Tissue Tek (Sakura Finetek, #1467); uPAR, F4/80, and CD3 IHC: After deparaffinization, the tissue sections were subjected to Ag retrieval by treatment with proteinase K (5 μg/ml) at 37°C for 15 and 10 min, respectively, for uPAR and F4/80. In the case of CD3, the slides were incubated in 10 mM citrate buffer (pH 6.0) at 98°C for 15 min. After washing, the samples were incubated in 1% H_2_O_2_ for 15 min for blocking of endogenous peroxidases, followed by subsequent washing in tap water and 0.5% TBS-T. Afterward, the slides were incubated with primary antibodies (rabbit anti-huPAR pAb at 1 μg/ml), rabbit anti-smuPAR pAb P47 at 0.5 μg/ml, anti-mouse F4/80 mAb (Abcam, #ab6640) at 0.1 μg/ml, and anti-mouse CD3 mAb (Dianova, #DIA-303) at 1:1000, all diluted in antibody diluent (Dako, #S3022) overnight at 4°C. Last, detection was performed with the following secondary antibodies, respectively, Envision–horseradish peroxidase (HRP) anti-rabbit (Dako, #4003) for uPAR, and VisUcyte-HRP anti-mouse antibody (R&D systems, #VC005-025) for F4/80 and CD3. Incubation was carried out for 45 min at RT before adding the chromogenic DAB substrate (Origene, #C09-12) for an additional 5 min. Sections were finally rinsed and counterstained with Mayer’s hematoxylin for 30 s, followed by dehydration, drying, and mounting, as formerly detailed. All stained sections were scanned by NanoZoomer-XR Digital slide scanner C12000-01(Hamamatsu) and analyzed with NDP.view2 Plus software.

### Immune profiling

The protocol for tissue dissociation and staining of immune cells and the applied gating strategy were adapted from (*77*–*79*). Briefly, the dissected tumors were gently rinsed with ice-cold 1× PBS, and any attached healthy tissues were removed using a sterile scalpel. The tumors were then finely minced with a razor blade into the wells of a six-multi-well plate on ice and enzymatically digested in 2 to 3 ml of digestion buffer [RPMI 1640 medium supplemented with 5 mM of CaCl2, Collagenase D (1.5 mg/ml; Roche, #11088858001), and deoxyribonuclease I (100 μg/ml)]. The samples were incubated for 35 min at 37°C, with gentle shaking every 10 min, to aid digestion. The dissociated single-cell suspensions were then rinsed with ice-cold media to neutralize enzymatic activity, filtered through a 70-μm cell strainer, and collected by centrifugation. Next, cell pellets were resuspended in 5 ml of ACK buffer (Gibco, #A1049201) for red cell lysis for 5 min at RT. After a washing step in ice-cold 1× PBS, the cells were counted and 2 × 10^6^ cells/100 μl were aliquoted to the wells of a 96-well round polypropylene bottom plate (Corning 96, #3879) and collected by centrifugation for subsequent staining. The live-dead stain was first performed by resuspending cell pellets in 100 μl of diluted Zombie Aqua Fixable dye (1:400) in 1× PBS, followed by a 20-min incubation in the dark at RT. A mixture of live and heat-killed splenocytes was included as a live-dead single-stained cell control (1:800). Immunostaining was then carried out using a general panel for lymphoid and myeloid cell discrimination. The following anti-mouse antibodies (purchased from BD Biosciences unless otherwise stated) were used: CD45-BUV395 (1:800), T cell receptor β–Pacific blue (BioLegend, 1:500), CD19–phycoerythrin (PE)–Cy7 (1:800), CD11b-PerCP-eFluor 710 (Invitrogen; 1:1600), Ly6C-PE (1:800), and Ly6G-allophycocyanin-H7 (1:400). Samples were stained using 100 μl of freshly prepared Ab master mixes in FACS staining buffer (SB, 1× PBS, 1% BSA), including True-Stain Monocyte Blocker (BioLegend, #426102) for Fc blocking, and incubated at 4°C for 20 min in the dark. Single-stained controls were prepared using UltraComp eBeads (Invitrogen, #01-2222-42). After surface staining and two washing steps with SB, samples and beads were fixed in 1% PFA in FASC buffer and stored at 4°C in the dark until detection. Samples were acquired on the Becton Dickinson LSRFortessa located in the UNC Flow Cytometry Core Facility. A minimum of 10^6^ and 10^4^ events per tube were collected for cell samples and beads, respectively. The FlowJo Software v10.8.1 was used for data analysis.

### Statistical analysis

Statistical relationships between data were determined by Pearson correlation analysis. Comparisons between two or multiple groups were analyzed via unpaired, two-tailed *t* test and one-way analysis of variance (ANOVA) (with post hoc Tukey’s correction), respectively, while the Mantel-Cox log-rank test was used for the Kaplan-Meier survival analysis. Statistical differences with *P* < 0.05 were considered significant and reported in the figure’s description. All analyses were conducted using GraphPad Prism 9 software.
