# Supplementary material for: Targeting uPAR with an antibody-drug conjugate suppresses tumor growth and reshapes the immune landscape in pancreatic cancer models
Source: Sci Adv. 2025 Jan 17;11(3):eadq0513. doi: 10.1126/sciadv.adq0513 (PMC11740940; doi:10.1126/sciadv.adq0513)
Supplement: Supplementary file 1 — Figs. S1 to S13 Table S1 [file sciadv.adq0513_sm.pdf]

Science Advances
AAAS

Supplementary Materials for

Targeting uPAR with an antibody-drug conjugate suppresses tumor growth and reshapes the immune landscape in pancreatic cancer models

Virginia Metrangolo *et al.*

Corresponding author: Lars H. Engelholm, lhe@finsenlab.dk;
Virginia Metrangolo, virginia.metrangolo@finsenlab.dk

Sci. Adv. **11**, eadq0513 (2025)
DOI: 10.1126/sciadv.adq0513

This PDF file includes:

Figs. S1 to S13
Table S1

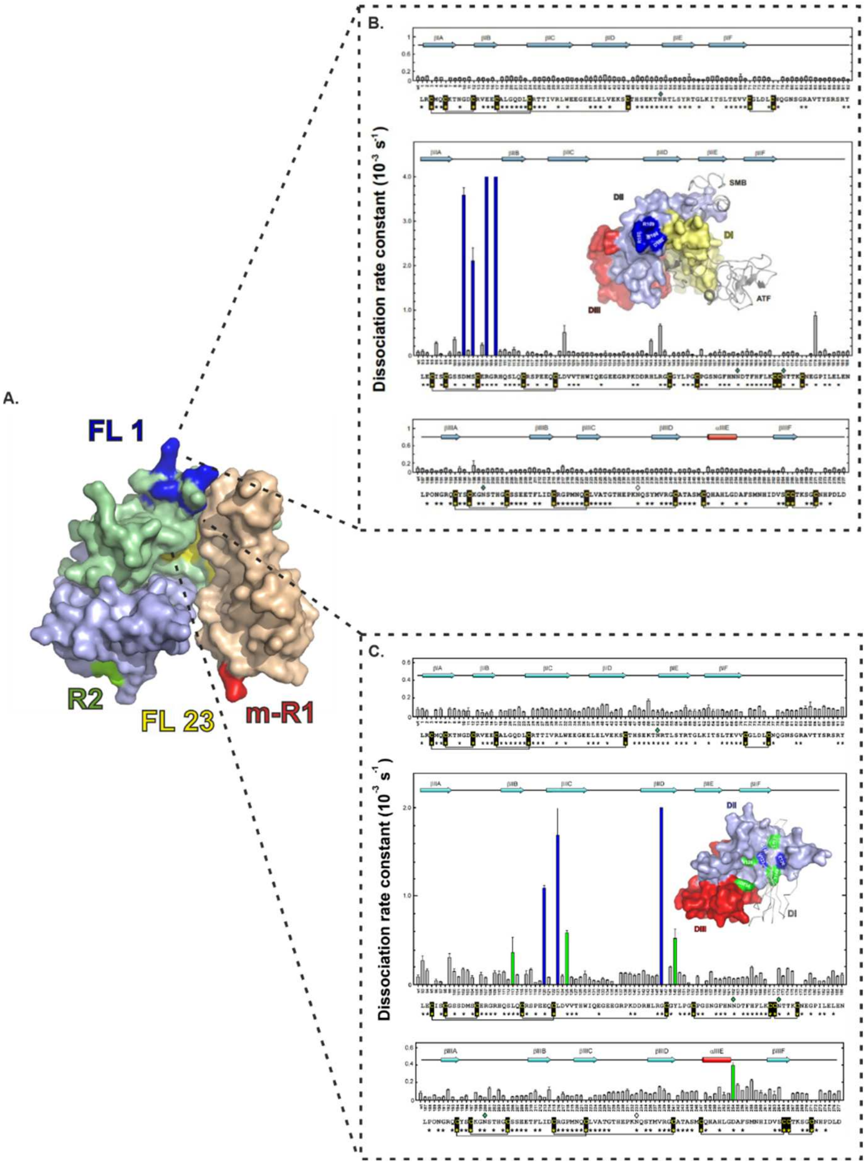

Fig. S1. The topographic epitope landscape on uPAR. (**A**) Surface representation of uPAR in its semi-open conformation (PDB code: 1YWH) with D1 green, DII light blue, and DIII wheat. The binding sites for FL1 (blue, DII), FL23 (yellow, DII), mR1 (red, DI), and R2 (green, DIII) epitopes are shown on a surface representation of uPAR. The epitope for FL23 is buried in the uPA binding cavity and becomes exposed when uPAR is in the open conformation. Detailed information of k_{off} values for uPAR complexes with FL1 (**B**) and FL23 (**C**) determined by SPR are plotted to delineate the respective epitopes. Secondary structure elements in uPAR are shown in the upper section and were inferred from the crystal structure [75]. The molecular models shown to the right in B and C highlight the location of the hotspots for FL1 and mR1. uPAR-Bound uPA (represented by ATF) and vitronectin (represented by SMB) are illustrated as ribbon diagrams.

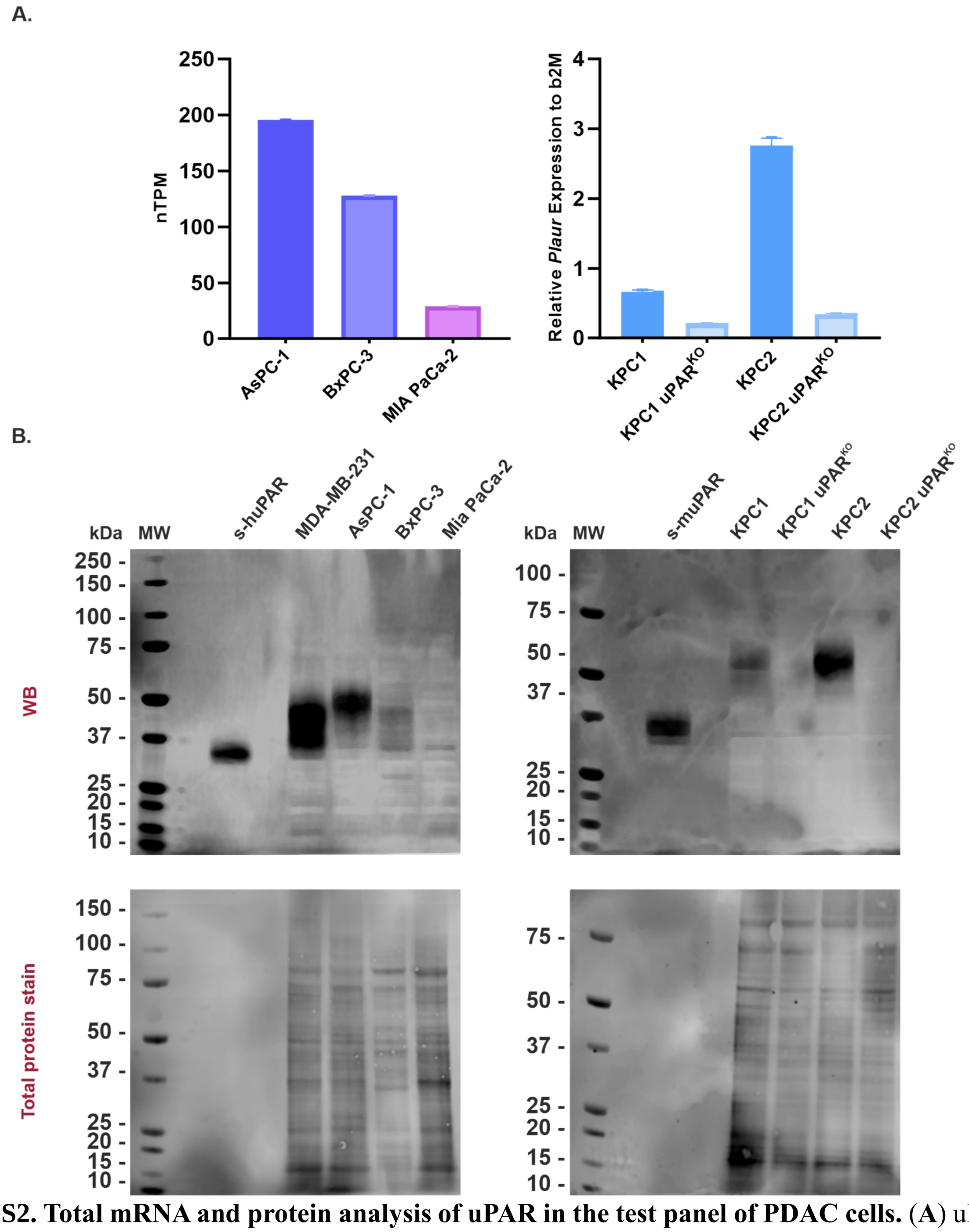

Fig. S2. Total mRNA and protein analysis of uPAR in the test panel of PDAC cells. (**A**) uPAR mRNA levels in human (left histogram, https://www.proteinatlas.org/ENSG00000011422-PLAUR/cell+line) and murine PDAC cells (right histogram, bars show the mean of biological triplicates ± SD), as measured by RT-qPCR. The cDNA used in this analysis was synthesized using 2 µg of RNA with High-Capacity RNA-to-cDNA™ Kit (Thermo Fisher Scientific, # 4387406), following the manufacturer's instructions. Quantitative RT-qPCR was then performed (using comparative Ct (ΔΔCt)) with the TaqMan™ Gene Expression Master Mix (Thermo Fisher Scientific, # 4369016) using probes for *Plaur* (Mm00440913_m1). All transcript levels were normalized to the housekeeping internal control B2m (Mm00437762_m1). Relative transcript

changes were determined using the Pfaffl method. nTPM, normalized transcripts per million. (**B**) Western Blot (WB) analysis of uPAR protein in whole cell lysates (35 µg) from human (left blot) and mouse (right blot) PDAC cells as determined by immunoblotting using anti-huPAR and anti-muPAR rabbit polyclonal mAbs, as specified in Material and Methods. The glycosylation of uPAR results in a characteristic electrophoretic profile consisting of a broad band of about 50-60 kDa, based on the cell line. Purified recombinant shuPAR and smuPAR (~ 37 kDa) produced in Drosophila S2-cells and the MDA-MB-231 (TNBC) lysate were included as positive controls, while the $uPAR^{KO}$ KPC cells served as negative controls, respectively. The more confined electrophoretic mobility of the soluble uPAR forms is due to their homogenous glycosylation with biantennary carbohydrates only, compared to intact uPAR (I-III) [74]. Equal protein loading was confirmed via Revert Total Protein Stain before immunoblotting (bottom panel). Membranes were imaged in the 700 and 800 nm channels using the Odyssey CLx fluorescence imaging system (LI-COR Biosciences).

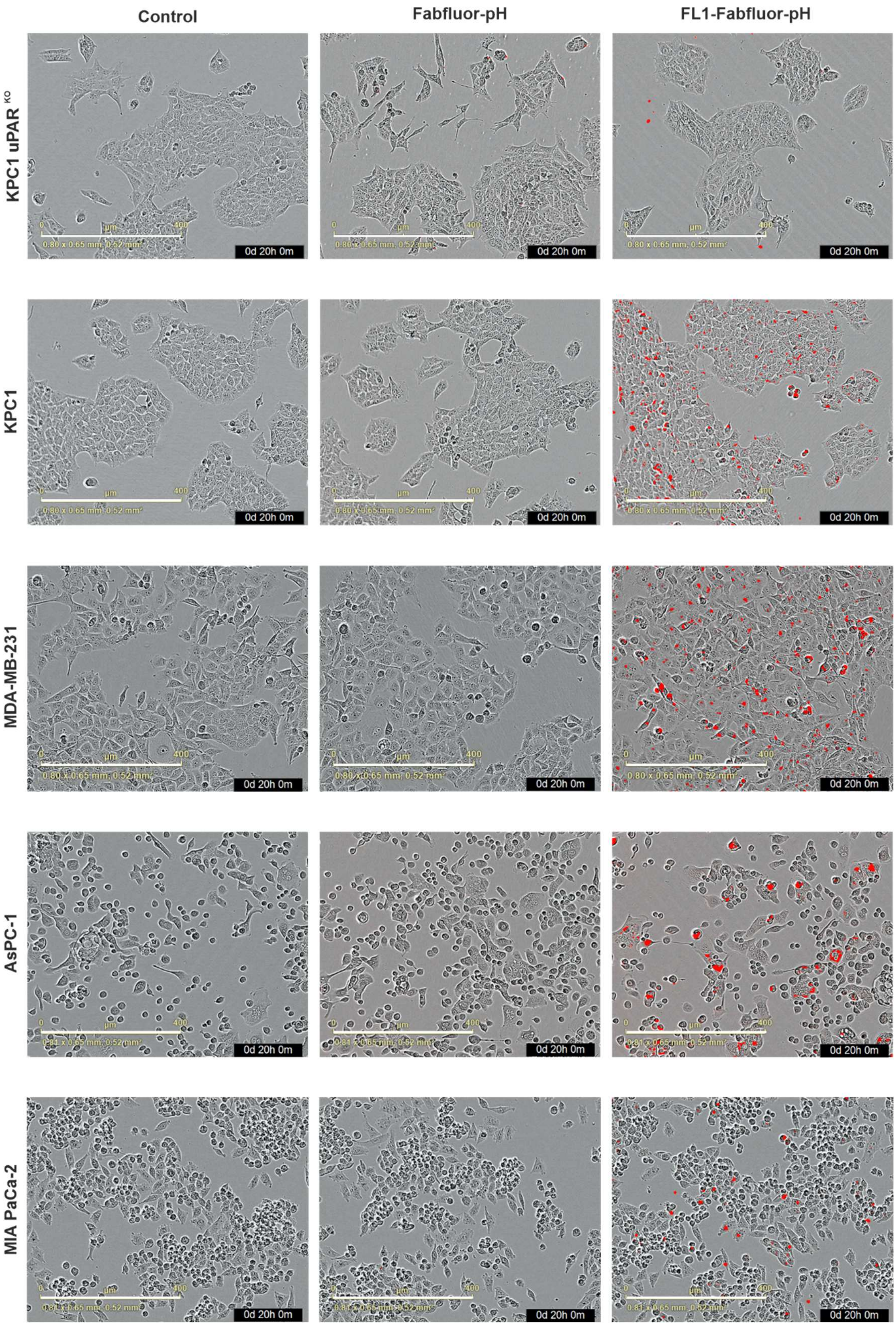

Control
Fabfluor-pH
FL1-Fabfluor-pH
KPC1 uPAR KO
KPC1
MDA-MB-231
AsPC-1
MIA PaCa-2
0d 20h 0m

Fig. S3. Phase contrast and red fluorescence images captured with the Incucyte® live-cell analysis system at 20 h (endpoint) of the internalization assay, supporting data in Fig. 2. For each cell line, representative images of control samples (unstained cells and cells incubated with Incucyte® Red Fabfluor-pH-dye alone) along with test samples (stained with the Incucyte® Red Fabfluor-pH-dye-labeled-FL1) are presented side-by-side. No red signal is detectable in the control samples of all analyzed cells, including the $uPAR^{KO}$ cells, confirming both receptor-specific endocytosis of FL1 and subsequent trafficking into the acidic organelles of the endocytic pathway. Scale bar, 400 μm.

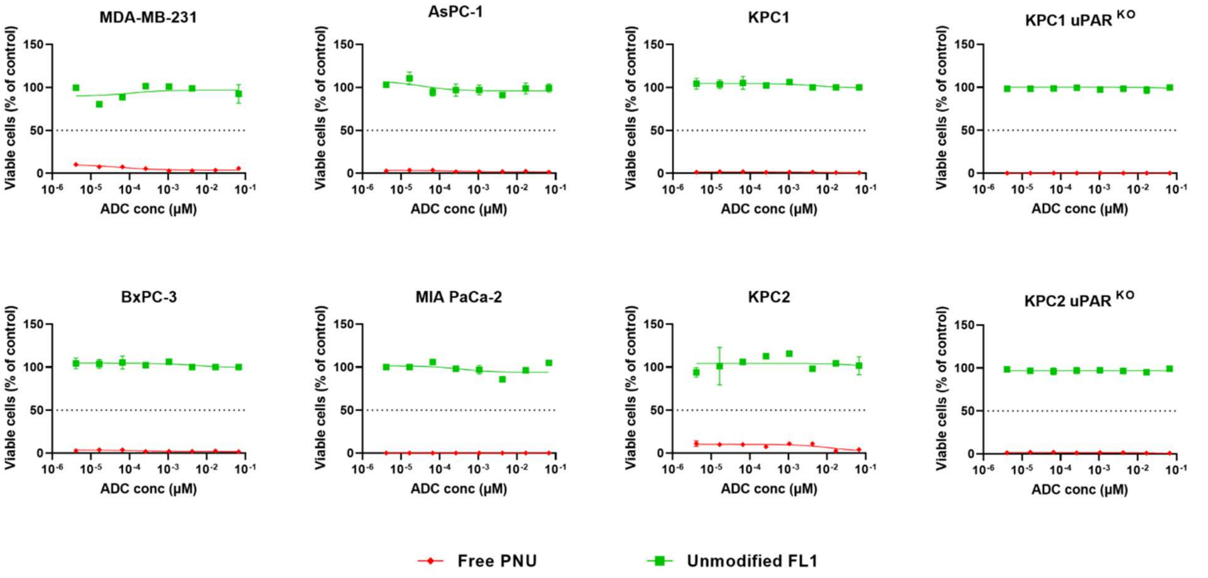

Fig. S4. ***In vitro*** **testing of free PNU and unmodified FL1.** Dose-response curves illustrating the *in vitro* sensitivity of free PNU and unmodified FL1, as assessed by MTS assay following 72 h of exposure. The percentage of cell viability relative to untreated control cells (n = 3, ± SD) is shown.

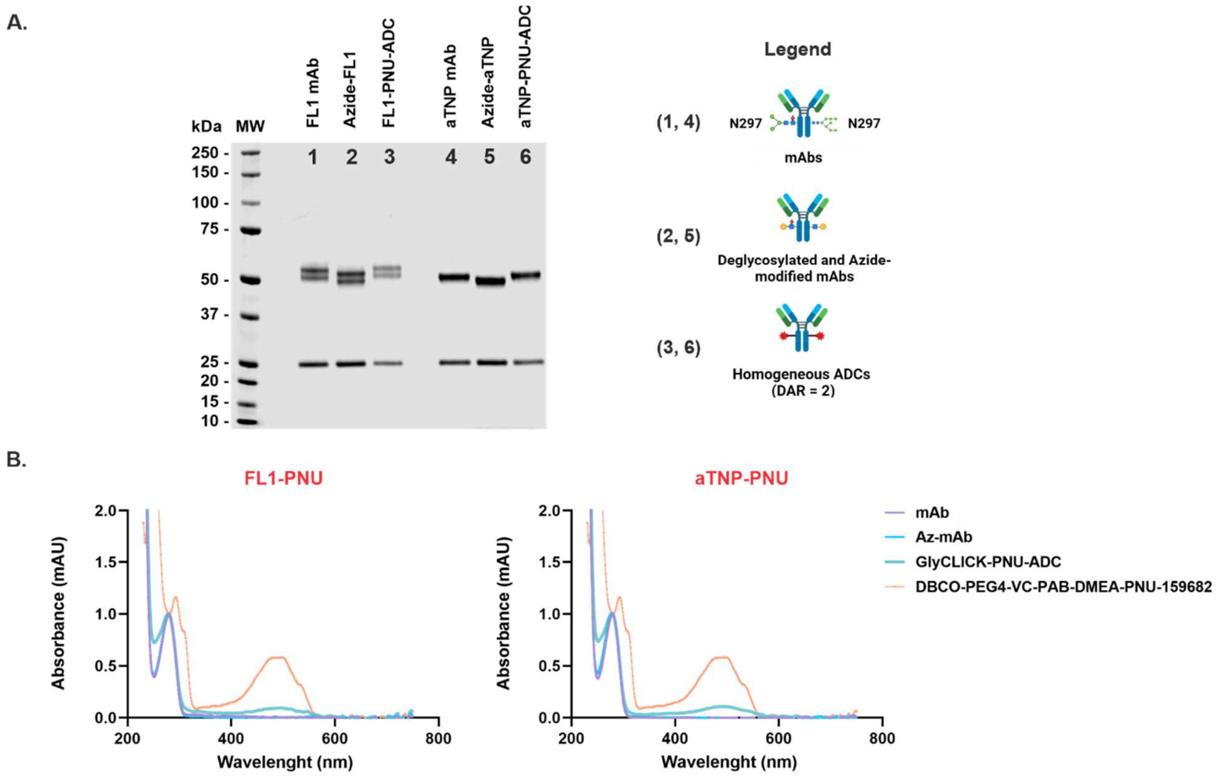

Fig. S5. Analytical characterization of mAbs and PNU-conjugates. (**A**) Coomassie staining of SDS-PAGE gels of reduced FL1 and negative control (NC) antibody (aTNP) and corresponding Azide-modified intermediates and final ADC conjugates using the GlyClick™ technology (supporting information to Fig. 3). The electrophoretic mobility of heavy chains (HC, 50 kDa) and light chains (LC, 25 kDa) bands are indicated. The first shift in electrophoretic mobility (lanes 2 and 5) corresponds to the enzymatic removal of the N-linked glycan (N297) in the HC. This deglycosylation is followed by azide (Az) activation and subsequent PNU conjugation (lanes 3 and 6). A legend with a schematic representation of the three reaction species is provided on the right. (**B**) DAR determination using UV-vis absorbance spectra of naked mAbs, Az-mAbs, ADCs, and free linker-payload. The molar DAR (expressed as the ratio of mAb/linker-payload concentrations) was calculated according to the Beer-Lambert law from separately determined absorbance and extinction coefficient values at 280 nm and 487 nm (A_{max} of the linker-payload). Calculated DAR for both ADCs matched the expected theoretical value of 2.

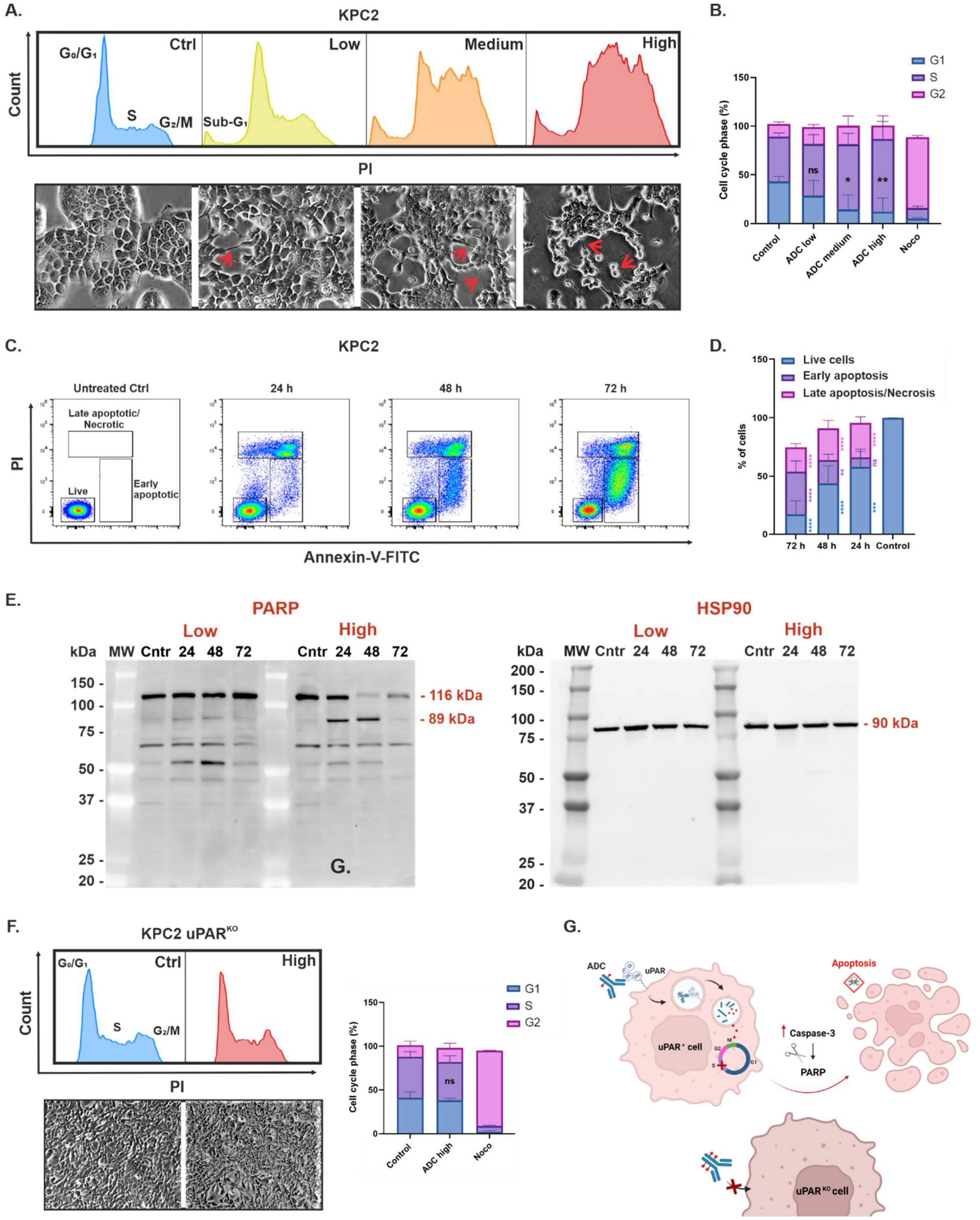

Fig. S6. Analysis of FL1–PNU mode of action *in vitro* and cell death kinetics. (**A**) The upper panel shows the cell cycle profile of KPC2 cells upon exposure to a 5-fold titration of FL1-PNU, starting from the relative EC_{50} value (0.02, 0.1, and 0.5 nM, herein referred to as low, medium,

and high concentrations). After 24 h incubation cells were stained with Propidium Iodine (PI) and the DNA-content distribution in each cell cycle phase was analyzed by flow cytometry. The anti-mitotic drug, Nocodazole, was used as a positive control of arrest in the G2 phase. In line with PNU-159682 DNA-damaging activity, a significant dose-dependent accumulation in the S-phase was observed in treated KPC2, relative to untreated control cells (from 46 % to 70%), at 0.1 nM (5X EC_{50}) with an almost complete arrest seen at the highest ADC concentration of 0.5 nM (25X EC_{50}) (from 46% to 79%). Besides, a sub-G1 peak, indicative of cells undergoing apoptosis, appeared at the EC_{50} value and increased gradually with the ADC concentration. A microscopic evaluation of the treated cells via a phase contrast microscope (10X), displayed in the lower panel, confirmed the appearance of typical apoptotic morphological changes, like shrinkage, apoptotic bodies, and monolayer disruption, which were more drastic at higher doses. (**B**) Stacked histogram indicating the estimated percentage of cells in each phase. (**C**) Time course study (24 h-72 h) of apoptosis in KPC2 cells treated with a fixed, high concentration of ADC (0.5 nM). Cells were double stained with anti-Annexin V–FITC and PI and the percentages of live (annexin V^-/PI^-), early (Annexin V^+/PI^-), and late apoptotic/necrotic (annexin V^+/PI^+) cells quantified by flow-cytometry, as presented in (**D**). Compared to untreated control cells, the ADC treatment significantly enhanced the proportion of both early and late apoptotic/necrotic cells. Overall, the total rate of apoptotic cells at 72 h was increased by 20 and almost 60%, compared to 24 h-treated and untreated cells, indicating a time-dependent apoptosis induction. (**E**) Representative immunoblotting (n = 3) of PARP (marker of DNA-damage-induced apoptosis) in lysates of FL1-PNU-treated KPC2 cells from the time course assay. This analysis (left panel) confirmed a time-dependent cleavage, already evident at 24 h, where the 119 kDa intact form and the cleaved 89-kDa fragment were both detected, compared to untreated control cells harboring the sole full-length protein. The cleavage appeared completed at 48 h, as the uncleaved protein band reappeared at 72 h. The process also seemed dose-dependent since the intact protein was predominant at all time points at EC_{50} values, while a dim cleaved fragment was partially visible. HSP90 (right panel) was included as a loading control. (**F**) Cell cycle distribution and micrographs of KPC2 $uPAR^{KO}$ cells treated with FL1-PNU at 0.5 nM showed no apparent changes compared to untreated control cells, confirming on-target cytotoxicity. (**G**) Schematic model of the proposed FL1-PNU MOA. All flow-cytometry data are representative of at least four independent experiments, and mean values ± SD are shown. Significant differences are indicated as follows: ns: non-significant; *, $p \leq 0.05$; **, $p \leq 0.01$; ***, $p \leq 0.001$; ****, $p \leq 0.0001$.

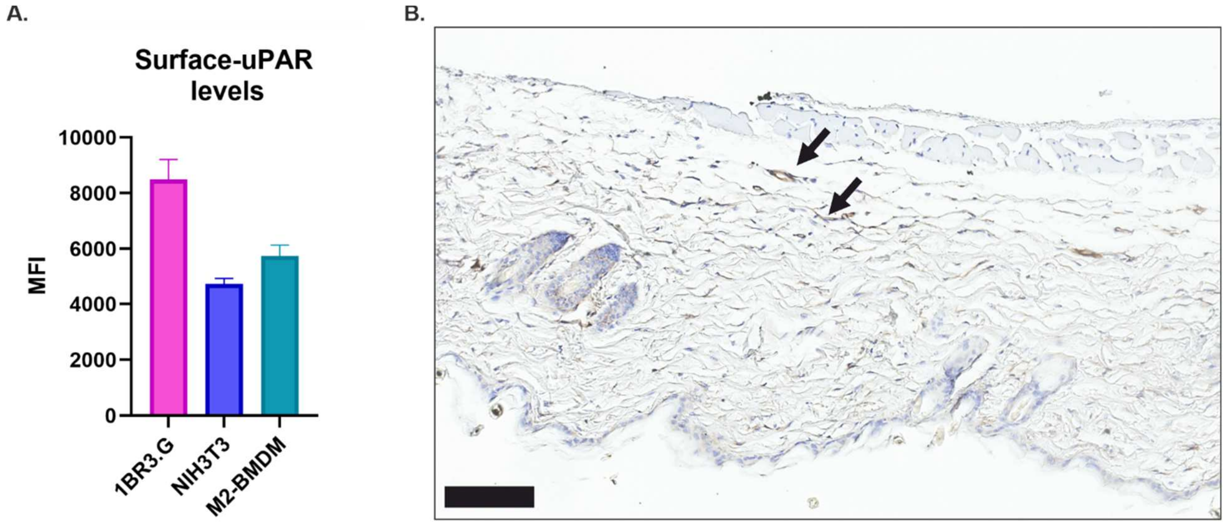

Fig. S7. Analysis of uPAR-expression in cultured fibroblasts and M2-polarized BMDM and normal mouse skin. (A) Flow cytometry analysis of surface uPAR-levels in human 1BR3.G, murine NIH3T3 fibroblast cells, and M2-differentiated BMDM. Bone marrow precursor cells were isolated from the pelvis, femur, and tibia of FVB/N mice (8-10 weeks), as outlined in (72). The collected cells were then differentiated to M2-like macrophages in 96-well plates (5 x 10^4 cells in 100 uL per well) in media (DMEM, 10% FBS, 1% P/S) containing 20 ng/mL mM-CSF (Biotechne) for four days, followed by an additional two days of stimulation with 20 ng/mL mM-CSF and 20 ng/mL mIL-4 (Biotechne). The flow cytometry analysis was conducted as described in Material and Methods. **(B)** Immunohistochemical staining of uPAR in normal mouse skin. Mouse skin from naïve C57BL/6 mice was immunostained for muPAR using a rabbit anti-smuPAR pAb P47 (as described in Material and Methods). Weak uPAR staining is detected in sparse quiescent fibroblasts in the skin (black arrows). Scale bar, 100 µm.

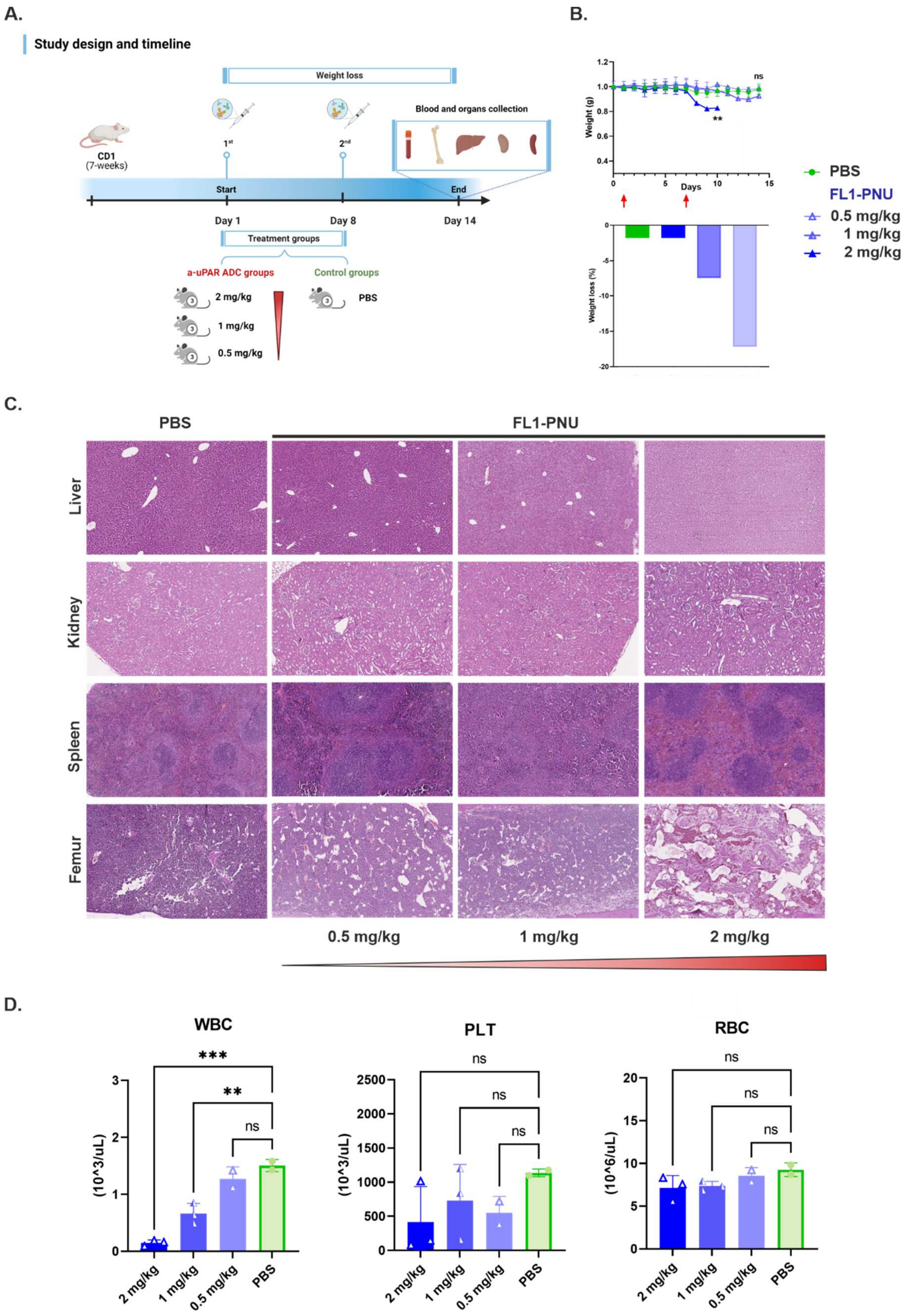
A.
Study design and timeline
Weight loss
Blood and organs collection
CD1
(7-weeks)
Start
End
Day 1
Day 8
Day 14
Treatment groups
a-uPAR ADC groups
Control groups
2 mg/kg
1 mg/kg
0.5 mg/kg
PBS
B.
Weight (g)
Days
ns
**
Weight loss (%)
PBS
FL1-PNU
0.5 mg/kg
1 mg/kg
2 mg/kg
C.
PBS
FL1-PNU
Liver
Kidney
Spleen
Femur
0.5 mg/kg
1 mg/kg
2 mg/kg
D.
WBC
PLT
RBC
(10^3/uL)
(10^6/uL)
***
**
ns
2 mg/kg
1 mg/kg
0.5 mg/kg
PBS

Fig. S8. Pilot study on drug tolerance in CD1 mice. (**A**) Experimental design and timeline. Animal tolerance towards FL1-PNU was assessed in a pilot dose scouting experiment in naïve CD1 mice. Treatment involved administration of three ADC doses at 0.5, 1, and 2 mg/kg under a weekly dosing schedule for two weeks. Animals were monitored for signs of distress or aberrant behavior and weighed daily, while blood and relevant organs were collected at the end of the study and examined. (**B**) Weight body curves over time (upper panel) and percentage loss (lower panel) at study termination relative to initial weight. (**C**) H&E staining of collected tissues and blood analysis (shown in **D**) revealed dose-dependent hematologic/bone marrow alterations consistent with the expected payload toxicity profile. Evident changes concerned white blood cell (WBC) and platelet (PLT) counts. Statistical significance was not achieved for the latter, likely due to biological variation and reduced animal number, but a clear trend is evident. **, $p \leq 0.01$; ****, $p \leq 0.0001$.

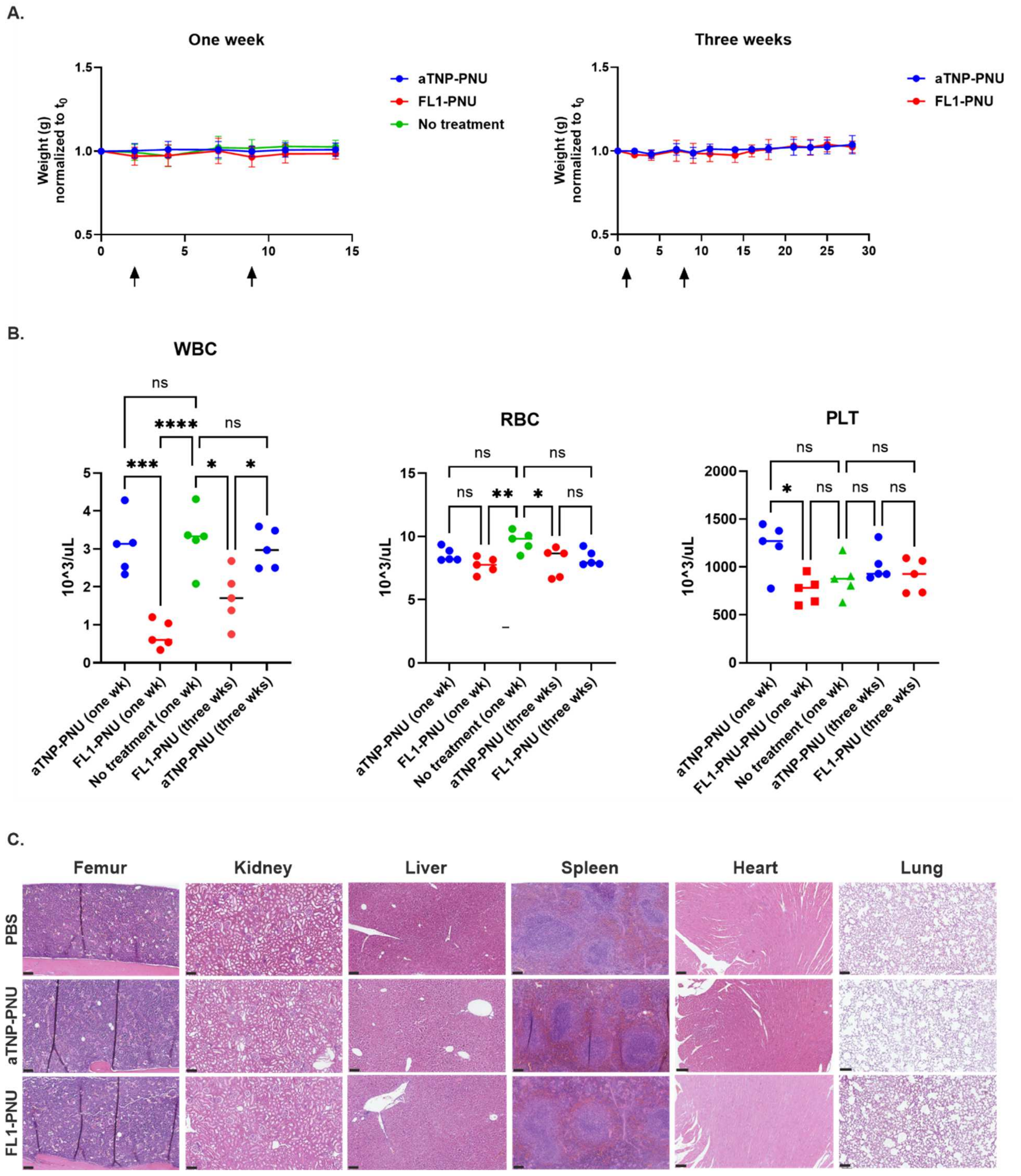

Fig. S9. Analysis of FL1-PNU tolerance at the refined dosage of 0.75 mg/kg in CD1 mice. (A) Animal tolerance towards FL1-PNU at 0.75 mg/kg was evaluated in naïve CD1 mice using the same dosage regimen as in Fig. S8. FL1- and aTNP-PNU ADCs were administered intravenously once a week for two weeks. To assess for acute effects and eventual recovery, mice in each treatment arm were randomized into two groups (n = 5) and sacrificed, respectively, one and three weeks after the last ADC administration. Untreated animals were included as a control and

terminated one-week post-treatment. Animal behavior and body weight were monitored every two days, while blood and vital organs, including, the femur, kidney, liver, spleen, heart, and lung, were collected and analyzed at termination. No significant weight loss was observed across the groups over the observation period. **(B)**. However, as expected and noticed in the initial pilot study (Fig. S8), acute hematological changes followed FL1-PNU treatment, with a significant reduction in total WBC and a slight decrease in PLT counts. Nevertheless, three weeks after treatment cessation, we observed almost complete hematological recovery, with PLT counts fully returned to baseline values. H&E staining revealed no obvious morphological changes in the major organs collected from each treatment arm compared to untreated animals. Scale bar, 100 μm. Significant differences are indicated as follows: ns: non-significant; *, $p \leq 0.05$; **, $p \leq 0.01$; ***, $p \leq 0.001$; ****, $p \leq 0.0001$.

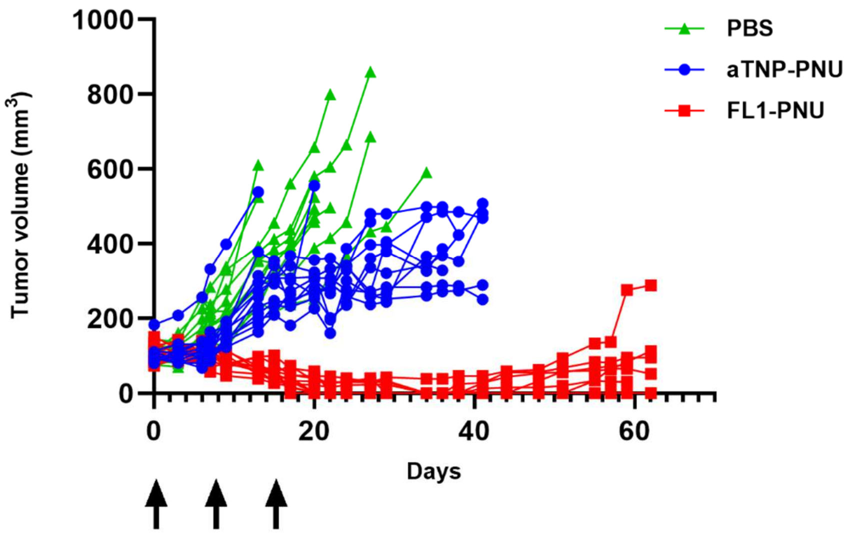

Fig. S10. Tumor growth curves of individual AsPC1-xenografted mice from each treatment arm. Compared to both control groups, three administrations of FL1-PNU induced complete remission or prolonged tumor regression in all but one of the twelve treated mice.

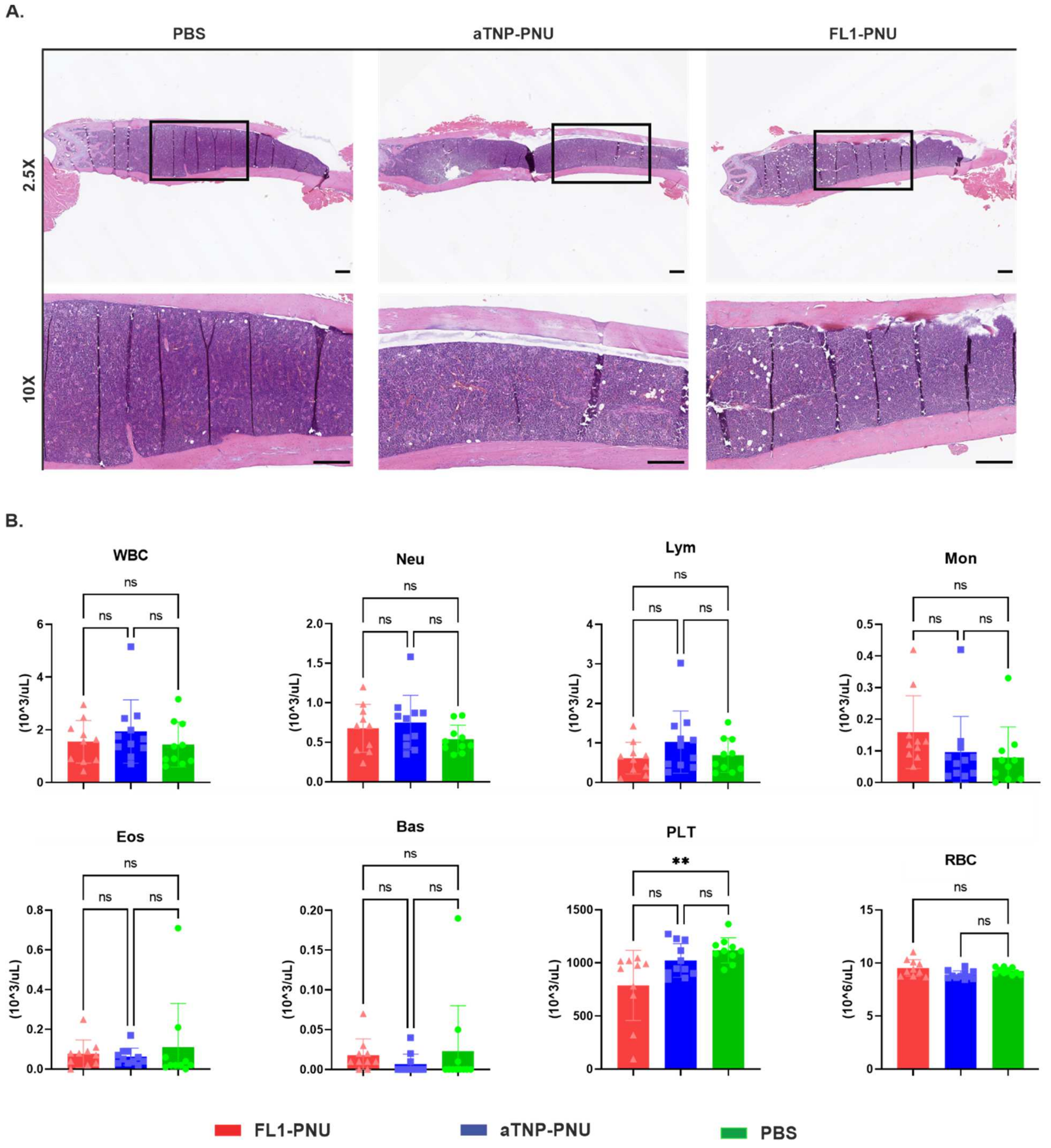

Fig. S11. Histological and hematological analysis of treated animals from the AsPC-1-derived xenograft study illustrated in main Fig. 5. (**A**) H&E staining of mouse femurs collected at study termination showing restoration of a normal bone marrow phenotype in the FL1-PNU treated animals. Scale bar, 400 µm. (**B**) Comprehensive hematological analysis revealed no significant differences between the three treatment groups, except for two cases of thrombocytopenia in the FL1-PNU-treated arm. **, $p \leq 0.01$.

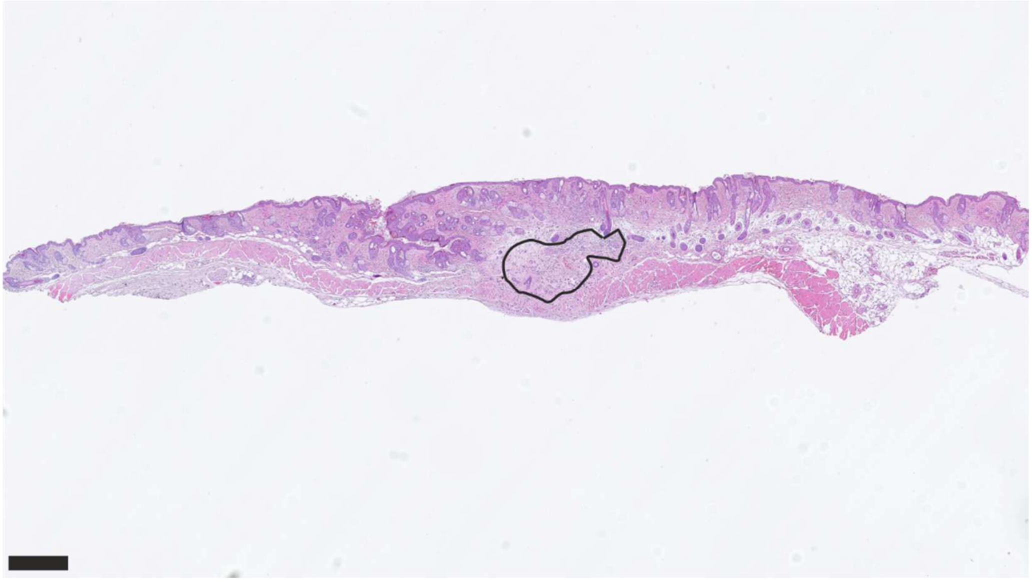

Fig. S12. Representative H&E staining of a resected tumor area from a cured AsPC-1-xenografted animal. The encircled area highlights residual tumor tissue in the excised region, surrounded by scar-like tissue. Scale bar, 500 µm.

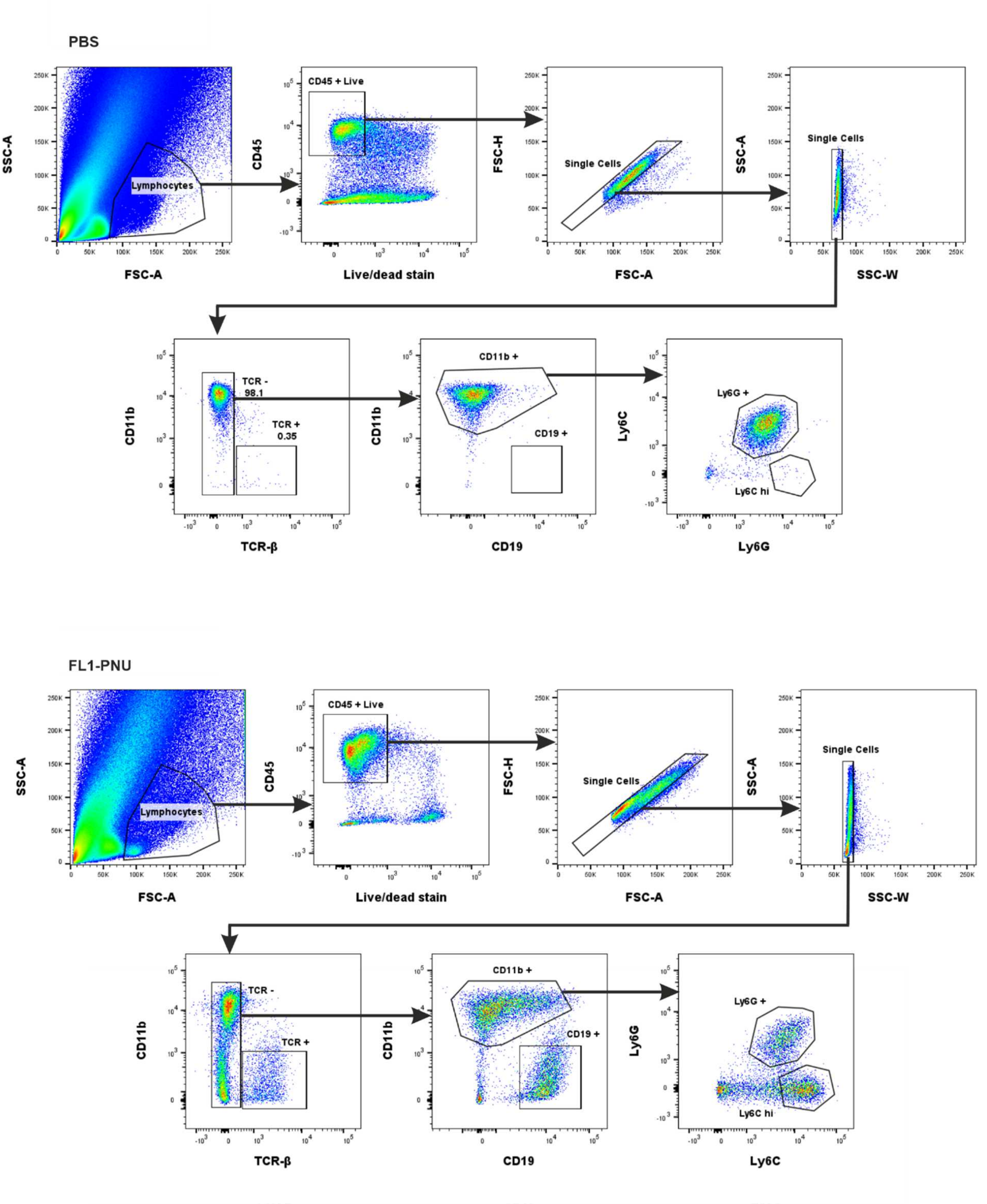

Fig. S13. Representative flow cytometry plots illustrating the gating scheme for the immune profiling in KPC2-derived orthotopic allografts displayed in Fig. 9. Leukocytes were gated according to morphology and size (SSC-A vs. FSC-A) and following doublets exclusion (FSC-H vs FSC-A), the live immune cells were selected using Zombie Aqua™ and CD45. A CD11b vs

TCRβ plot was used to discriminate between TCR^+ T cells and TCR^- cells. A subsequent CD11b vs CD19 plot was then applied to differentiate myeloid cells ($CD11b^+$, $CD19^-$) from B cells ($CD19^+$, $CD11b^-$). The former population was further dissected into neutrophils/PMN-MDSCs ($CD11b^+$, $Ly6G^+$, $Ly6C^{int}$) and mono/mø cells ($CD11b^+$, $Ly6G^-$, $Ly6C^+$ and $Ly6C^{low/-}$). Of the latter, the Ly6C+ subset was finally identified as inflammatory mono/mø.

mAb	uPA inhibition	Epitope	Subtype	Cross-reactivity	Affinity K_D (nM)	Ref.
**FL1**	no	**D2** (Ser^{100}, Asp^{102}, Ser^{104}, Arg^{107}, Arg^{109})	IgG_1, κ	Yes (human and mouse)	0.24 (huPAR) 2.32 (muPAR)	This work
**mR-1**	yes	**D1** (Leu^{19}, Asp^{22})	IgG_1, κ[6]	Yes (human and mouse)	0.54 (huPAR) 5.2 (muPAR)	[68, 71]
**R2**	no	**D3** (Asp^{275}, Leu^{276})	IgG_1, κ	No (human)	0.02	[68, 69]
**R4**	no	**D2D3** (Arg^{192}, Asp^{214}, Gly^{217}, Ser^{269})	IgG_1, κ	No (human)	0.62	[68, 69]
**R8**	no	**D2D3** (Arg^{192}, Asp^{214}, Gly^{217}, Ser^{269})	IgG_1, κ	No (human)	0.43	[68, 70]
**FL23**	yes	**D2** (Leu^{113}, Glu^{120}, Leu^{123}, Val^{125}, Gly^{146}, Tyr^{149}, Asp^{254})	nd	No (human)	0.57	This work

Table S1. Binding affinity and epitopes of the investigated a-uPAR monoclonal antibodies. Epitopes on uPAR for the various mAbs were mapped by surface plasmon resonance (SPR) studies using a complete alanine-scanning library of soluble uPAR mutants (as described in [68, 73]) and are shown in Figure S1. The binding affinities and epitopes for FL23 and FL1 are presented for the first time in this work. FL1 and mR-1 display cross-reactivity towards human and mouse uPAR and were obtained by immunizing uPAR-deficient mice with recombinant purified soluble human and mouse uPAR, respectively.
